# Deepening the Modulatory Activity of Bioactive Compounds Against AFB1- and OTA-Induced Neuronal Toxicity Through a Proteomic Approach

**DOI:** 10.3390/antiox14050571

**Published:** 2025-05-09

**Authors:** Alessandra Cimbalo, Massimo Frangiamone, Lara Manyes

**Affiliations:** Biotech Agrifood, Faculty of Pharmacy and Food Sciences, Universitat de València, Avda. Vicent Andrés Estellés s/n, 46100 Burjassot, Spain; massimo2.frangiamone@uv.es (M.F.); lara.manyes@uv.es (L.M.)

**Keywords:** bioactive peptides, carotenoids, proteomics, SH-SY5Y, HPLC-MS/MS-QTOF

## Abstract

The aim of this work is to highlight the beneficial effects of bioactive peptides present in fermented whey (FW) and carotenoids from pumpkin (P) against the pro-oxidant effects of aflatoxin B1 and ochratoxin A at the neuronal level. For this purpose, SH-SY5Y human neuroblastoma differentiated cells were exposed to (A) mycotoxins, (B) the digesta of mycotoxin-contaminated bread formulated with P, or (C) bread enriched with FW + P. A proteomic approach using HPLC-MS/MS-QTOF was then employed to characterize the metabolic pathways affected by the presence of these components, as well as their ability to modulate the toxic effects exacerbated by mycotoxins. Gene ontology functional analysis revealed proteins primarily associated with nucleosome structure, such as the H3-H4 tetramer, H2A-H2B dimer, and HIRA, which were overexpressed in the presence of mycotoxins and, interestingly, downregulated with the addition of the functional ingredients. Additionally, important metabolic pathways associated with the RHO GTPase family, estrogen-dependent gene expression, and androgen receptor transcription stimulated by PKN1 activation were discovered. Network interaction analysis highlighted the modulation of cytoskeletal dynamics, cell migration, and stress responses. These findings provide novel insights into the neuroprotective potential of functional food components, supporting their use in mitigating mycotoxin-induced neuronal damage and opening new avenues for dietary-based neuroprotection strategies.

## 1. Introduction

Mycotoxins, such as aflatoxin B1 (AFB1) and ochratoxin A (OTA), are toxic secondary metabolites produced by various species of fungi, mainly belonging to the genera *Aspergillus* and *Penicillium*. These compounds can contaminate a wide range of agricultural products and food supplies, especially cereal and cereal-based products, posing a significant issue for human health [1]. Indeed, once absorbed, AFB1 is metabolized in the liver with the production of a reactive epoxide, which is the major contributor to hepatotoxicity, nephrotoxicity, immunotoxicity, and carcinogenesis in human and animal models. Likewise, OTA has been reported to promote nephrotoxicity and immune toxicity in vitro and in vivo [2]. In addition to organ-specific toxicities, both AFB1 and OTA have also been implicated in neurological damage, particularly in relation to neuronal differentiation, raising potential threats to brain development and cognitive function [3]. In particular, AFB1 and OTA, through their ability to cross the blood–brain barrier and induce oxidative stress and inflammation, along with impairment of cellular signaling pathways, have been shown to interfere with the normal cell differentiation, thereby compromising brain health [4,5].

In light of this, exploring protective strategies against AFB1- and OTA-induced neurotoxicity has become an important area of research. For instance, functional foods and natural bioactive compounds have emerged as promising candidates for mitigating mycotoxin effects on neuronal health [6]. Pumpkin (P) (*Cucurbita* spp.) and fermented whey (FW) are two bioactive-rich food products showing antioxidant, anti-inflammatory, and neuroprotective properties. Pumpkin is known for its high content of carotenoids, polyphenols, and vitamins, which can protect against oxidative damage and support neuronal function [7]. Fermented whey, a byproduct of cheese production, is rich in bioactive peptides, probiotics, and amino acids, which enhance its neuroprotective potential [8]. Both of these food products have been explored for their ability to counteract various forms of cellular damage, but their effects against AFB1- and OTA-induced alterations in neuronal differentiation remain understudied [9].

In this context, it is important to mention how neuronal differentiation is a critical step in brain structure and function. Alterations in this process can lead to severe neurodevelopmental disorders, cognitive impairments, and may contribute to neurodegenerative diseases later in life [10]. Although the study of this process is fairly complicated, the use of SH-SY5Y human neuroblastoma cells undergoing differentiation has greatly simplified the analysis of cellular structures and events of great importance in neuroscience [11,12]. SH-SY5Y cells, with a high similarity to adult human neurons, represent a realistic, reproducible, readily available, and inexpensive model to study mycotoxin neurotoxicity in vitro [13].

While several technologies can be used in the field of toxicological research, omics techniques have proven to be one of the most all-embracing and valuable [14]. In detail, proteomics, the large-scale study of proteins, provides a powerful tool for understanding the molecular mechanisms underlying the effects of AFB1 and OTA on neuronal differentiation, as well as the potential protective role of pumpkin and fermented whey [15]. Analyzing protein expression profiles can offer insights into the specific pathways and cellular processes that are disrupted by mycotoxins and identify how bioactive compounds can restore normal brain function. In addition, key proteins involved in neurodevelopment, oxidative stress response, and cellular signaling pathways can be also detected and subsequently used for future applications [16,17].

In the present study, the research goal was to investigate the protective effects of P and FW against AFB1- and OTA-induced alterations in neuronal differentiation in vitro by using a proteomic evaluation. Furthermore, to mimic a plausible scenario, a human in vitro digestion system was used to obtain intestinal bread extracts contaminated with AFB1 and OTA individually and in combination as well as with P and FW.

## 2. Materials and Methods

### 2.1. Reagents

DL-Dithiothreitol (DTT), with a purity of ≥99.0%, Trizma^®^ hydrochloride (Tris-HCl) for protein extraction, also ≥99.0%, and trypsin for protein digestion were sourced from Sigma Aldrich (St. Louis, MO, USA). Iodoacetamide (IAA), with a purity of 98%, was procured from ACROS Organics™, Thermo Fisher Scientific (Bridgewater, NJ, USA). Thiourea (TU), 99%, for the preparation of lysis buffer, was obtained from Thermo Fisher Scientific (Kandel, Germany), while Urea (U), 99%, was acquired from F.E.R.O.S.A (Barcelona, Spain). For QTOF analysis, acetonitrile (ACN) of LC/MS-grade OPTIMA^®^ (≥99.9% purity) was supplied by Fisher Chemical (Geel, Belgium). Formic acid (≥98%) was sourced from Sigma Aldrich (St. Louis, MO, USA). Deionized water, with a resistivity of less than 18 Ω·cm, was produced using a Milli-Q water purification system (Millipore, Bedford, MA, USA). The reagent-grade chemicals and cell culture compounds used were DMEM Ham’s-F12, penicillin, trypsin/EDTA solutions, fetal bovine serum (FBS), and phosphate buffer saline (PBS) from Sigma Chemical Co. (St. Louis, MO, USA). The standard solution stock (purity: 99%) of AFB1 and OTA, along with phosphate buffer saline (PBS), was obtained from Sigma-Aldrich (St. Louis, MO, USA). All stock solutions (1000 mg/L) were prepared by dissolving 1 mg of mycotoxin in 1 mL of pure methanol. These stock solutions were subsequently diluted with methanol to achieve the desired multi-compound working standard solutions, which were stored at −20 °C.

The natural ingredients for bread preparation, including pumpkin, wheat flour, mineral water, salt (NaCl), and sugar (sucrose), were sourced from a local supermarket in Valencia, Spain. Goat milk whey, coagulated using commercial rennet (starter culture R-604), was purchased from ALCLIPOR (Benassal, Spain). The lactic acid bacteria (LAB) used in this study, Lactobacillus plantarum CECT 220, was obtained from CECT (Paterna, Valencia, Spain). For in vitro digestion: Potassium chloride (KCl), potassium thiocyanate (KSCN), sodium dihydrogen phosphate (NaH_2_PO_4_), sodium sulfate (Na_2_SO_4_), sodium chloride (NaCl), sodium bicarbonate (NaHCO_3_), urea (CO(NH_2_)_2_), α-amylase (930 U mg^−1^ A3403), hydrochloric acid (HCl), sodium hydroxide (NaOH), formic acid (HCOOH), pepsin A (674 U mg^−1^ P7000), pancreatin (762 U mg^−1^ P1750), bile salts (B8631), and phosphate-buffered saline (PBS, pH 7.5) were all acquired from Sigma-Aldrich (Madrid, Spain).

### 2.2. Flour Contamination, Bioactive Ingredients, and Bread Preparation

The procedure for flour contamination and bread preparation follows the protocol established by [18]. Barley and maize flour were naturally contaminated by the fungal species producers of AFB1 and OTA, *Aspergillus steynii* 20510 (obtained from CECT) and *A. flavus* ITEM 8111 (obtained from the Agro-Food Microbial Culture Collection of The Institute of Sciences and Food Production (ISPA, Bari, Italy)), respectively. For this purpose, 300–350 g of maize or barley were placed in 1 L autoclaved glass jars. The cereals were then inoculated with 15–20 mL of a spore and mycelium suspension in peptone water with Tween 80 (0.1% both) of the corresponding fungal strain. The jars were incubated at room temperature in the dark for one month. After incubation, cereals were autoclaved to inactivate the fungi and subsequently ground into flour until fully homogenized.

Bioactive ingredients were prepared as follows. Milk whey, derived from goat milk coagulated with commercial rennet (starter culture R-604), was provided by ALCLIPOR society, S.A.L. (Benassal, Spain). Although both fermented and non-fermented versions were initially prepared, only the fermented milk whey was used in this study. For fermentation, 4 mL of lactic acid bacteria (LAB) suspension (10^8^ CFU/mL) were added to 40 mL of pasteurized milk whey, followed by incubation at 37 °C for 72 h. The resulting product was then lyophilized to obtain a homogeneous powder. Notably, the antioxidant activity of this LAB-fermented whey, produced by our research group, has been previously analyzed and described by [19]. Additionally, pumpkin was obtained from a local supermarket in Valencia (Spain). The samples, identified as *Cucurbita maxima* (Hokkaido variety), were peeled, deseeded, lyophilized, and ground into a fine powder. These samples have been previously characterized from various perspectives in earlier studies [20,21]. Both the pumpkin and the fermented whey powders were analyzed to confirm the absence of mycotoxins and stored at −20 °C until use.

For the experiment, eight loaves of bread were prepared by combining mycotoxins (AFB1, OTA) with bioactive ingredients (Table 1). Briefly, the control bread was prepared using the following recipe: 300 g of wheat flour, 165 mL of mineral water (at 37 °C), 20 g of bakery yeast (Levital, Spain), 10 g of sucrose, and 6.5 g of NaCl. After combining the ingredients, the dough was mixed in a bakery machine (Silver Crest) for 5 min, then shaped into 100 g portions and placed in molds. The loaves were covered with a damp cloth and left to ferment at room temperature for 1 h. Next, the breads were wrapped in silver foil and baked at 200 °C for 45 min in a Memmert ULE 500 muffle furnace (Madrid, Spain). After baking, the breads were removed from the molds and cooled at room temperature for 1 h. Contaminated breads were prepared by substituting a portion of wheat flour with contaminated flours: 1.2 g of OTA-contaminated barley flour and/or 10 g of AFB1-contaminated maize flour, depending on the condition. For the enriched breads, modifications were made to the control recipe by adding 1% fermented milk whey, 1% lyophilized pumpkin, or a combination of 1% fermented milk whey and 1% lyophilized pumpkin.

### 2.3. In Vitro Digestion Model

The in vitro gastrointestinal digestion of the bread samples was performed following a protocol adapted from INFOGEST guidelines [22] and based on previously reported methods [19,23], with slight modifications. A 10 g portion of each test material was initially mixed with simulated salivary fluid (SSF) composed of both inorganic and organic components. The inorganic phase contained KCl (89.6 g L^−1^), KSCN (20 g L^−1^), NaH_2_PO_4_ (8.8 g L^−1^), Na_2_SO_4_ (57 g L^−1^), NaCl (175.3 g L^−1^), and NaHCO_3_ (84.7 g L^−1^), while the organic phase included urea (25 g L^−1^), α-amylase (290 mg), and mucin (25 mg) in a final volume of 500 mL, with pH adjusted to 6.8 ± 0.2. A defined volume (6 mL) of the prepared saliva was added to each sample to simulate the oral phase.

The gastric phase was initiated by adjusting the pH to 2.0 using 6 M HCl, followed by the addition of freshly prepared porcine pepsin (0.05 g/g of sample, corresponding to approximately 2000 U/mL, in accordance with INFOGEST recommendations). Milli-QPLUS H_2_O (Merck, Rahway, NJ, USA) was used to adjust the final weight to 100 g for fresh samples and 200 g for freeze-dried samples, reflecting their differing hydration capacities. Samples were incubated at 37 °C for 2 h with gentle orbital shaking (250 rpm).

To simulate the transition to the intestinal phase, the pH was raised to 6.5 ± 0.2 using 1 N NaHCO_3_. A pancreatin/bile salt mixture was then added (0.1 g pancreatin and 0.625 g bile salts dissolved in 25 mL of 0.1 N NaHCO_3_), and the samples were further incubated for 2 h at 37 °C under the same conditions. After incubation, the pH was adjusted to 7.2 ± 0.2 using 0.5 N NaOH. The digested samples were centrifuged at 4500 rpm for 10 min at 4 °C, and the supernatant was filtered through a 0.22 μm membrane. Aliquots of the intestinal digest were either directly injected into the HPLC-MS/MS-QTOF system for mycotoxin analysis or frozen (−80 °C) and subsequently lyophilized for storage at −20 °C. All digestion experiments were performed in triplicate to ensure reproducibility.

### 2.4. Cell Culture, Differentiation Protocol, and Experimental Setup

SH-SY5Y human neuroblastoma cells were selected for their ability to differentiate into neuron-like cells under retinoic acid stimulation, allowing a physiologically relevant model for neurotoxicity assessment [24]. Cells were cultured in DMEM/F12 medium supplemented with 10% FBS, 100 U/mL penicillin, and 100 mg/mL streptomycin under standard incubator conditions: pH 7.4, 5% CO_2_ at 37 °C, and 95% air with constant humidity. The culture medium was changed every 2–3 days. Herein, differentiation was induced over 7 days by supplementation with 10 μM All-Trans-Retinoic-Acid (RA), added at 0.1% (v/v) in differentiation medium (DMEM-F12 with 100 U/mL penicillin, 100 mg/mL streptomycin and 1% FBS).

**Figure 1:**
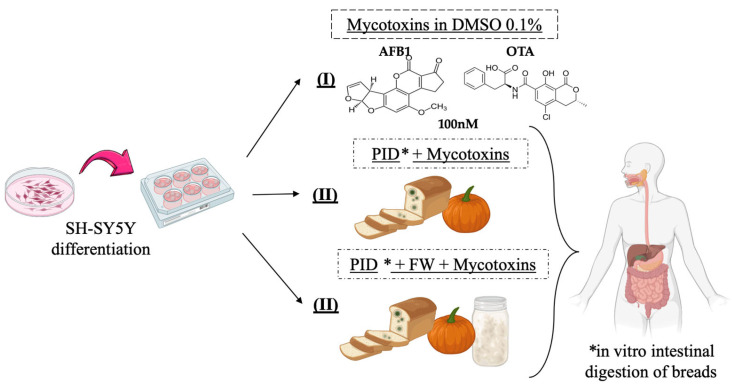
Experimental in vitro design of SH-SY5Y cells.

The in vitro experimental design carried out in this study consisted of exposing differentiated SH-SY5Y cells to three distinct batches of conditions during 7 days: (I) one to a standard mycotoxin (AFB1, OTA, AFB1 + OTA) concentration of 100 nM in 0.1% DMSO, (II) a second to intestinal digests of bread prepared with lyophilized pumpkin (PID) combined with mycotoxins, (III) and a third with the digest containing both functional ingredients (PID + FW) and mycotoxins (Figure 1). The culture medium and mycotoxin treatment were changed every 48 h.

The digested bread extracts were previously prepared as described in Section 2.3. After preparation, the extracts were centrifuged using a benchtop centrifuge (Eppendorf, 5804R, Hamburg, Germany) at 4000× *g* for 20 min at 4 °C using 50 mL conical tubes to remove any residual solid particles. The resulting supernatant was carefully collected and diluted 1:50 with the differentiation medium prior to use in cell treatments. The choice of this dilution is based on the idea of preventing the possible cytotoxicity of digested bread extracts (indeed, a 1:10 dilution of the extracts significantly reduced cell viability) and also to mimic the amount of mycotoxin that can cross the blood–brain barrier and reach the brain [5]. Moreover, it was determined based on prior analytical quantification.

### 2.5. Cell Viability Assay

The assessment of cell viability after treatments was conducted using the Trypan blue viability assay. For this purpose, cells were centrifuged for 5 min at 1400 rpm, and the resulting pellet was resuspended in 1 mL of culture media. A mixture was prepared by combining 10 μL of the resuspended cells with 10 μL of a commercially available trypan blue dye solution (Sigma-Aldrich, Buchs, Switzerland). The cells were then counted using an automatic cell counter, Countess™ 3 (Invitrogen™, Madrid, Spain). Each sample was analyzed in triplicate, and the means and standard deviations were calculated for all conditions. One-way ANOVA (GraphPad Prism 10.1.1) was employed to determine significant differences compared to the control, with a significance threshold set at *p* ≤ 0.05.

### 2.6. Gastrointestinal Extracts Analysis

#### 2.6.1. Mycotoxin

For mycotoxin quantification in intestinal extracts, they were directly filtered (0.22 μm filter) and injected in the HPLC-MS/MS-QTOF system. Standard calibration curves were prepared in methanol (1–1000 ng/mL) from OTA and AFB1 standards (1000 ng/mL). For quantitation purposes, matrix-matched calibration curves were prepared for all conditions by spiking blank digested extracts with OTA and AFB1 at the same concentrations.

#### 2.6.2. Phenolic Content

The extraction of phenolic compounds was carried out using the method described by [25], with some modifications. Five milliliters of intestinal digest were mixed with a solution of 1% formic acid in ethyl acetate (1:1), 2 g of MgSO_4_, and 50 mg of NaCl using a VWR International vortex (Barcelona, Spain) for 2 min. The extract was centrifuged at 3000 rpm (Eppernord AG 22331, Hamburg, Germany) for 10 min, and the supernatant was shacked for 2 min with 75 mg of C18 and 600 mg of MgSO_4_. The samples were centrifuged again at 3000 rpm for 10 min, and the supernatant was completely dried under a continuous nitrogen flow (Turbovap LV, Zymark Runcorn, UK). Finally, the sample was resuspended in 1 mL of water: acetonitrile (90:10 *v*/*v*), filtered through a 0.22 μm nylon filter (Phenomenex, Torrance, CA, USA), and stored for chromatographic analysis.

#### 2.6.3. Carotenoids Content

Carotenoids were extracted from the digest as described by [26]. In brief, each sample was extracted using a hexane/acetone mixture (3:2 *v*/*v*) directly in centrifuge tubes through a vortexing–sonication method. Specifically, the samples were vortexed for 1 min, followed by sonication for 1 min at a frequency of 40 ± 5 kHz. The extraction procedure was carried out twice, and the resulting organic phases were combined. These phases were then washed with 2 mL of 0.1 M NaCl, dried under a gentle nitrogen stream, and stored at −80 °C until LC-MS analysis. Prior to MS analysis, the dried extracts were re-dissolved in a solution of methanol: Methyl Tert-Butyl Ether (MTBE) (0.1% BHT) (1:1, *v*/*v*), filtered through PTFE filters (0.45 μm pore size), and analyzed using LC-MS/MS.

### 2.7. Protein Extraction and Sample Preparation for Proteomics

After exposure, the cells were harvested and washed with deionized water to eliminate residual medium and adjust the pH to neutrality. Cell pellets were lysed in buffer (8 M U/2 M TU/50 mM Tris-HCl) with repeated vortexing and sonication cycles and solubilized (USC 1200D ultrasonicator, VWR, International bvba, Leuven, Belgium) in iced methanol: H_2_O solution (1:1) to ensure complete protein solubilization. The lysate was centrifuged (13,000× *g*, 30 min, 4 °C) and the supernatant, containing solubilized proteins, was collected.

Protein concentration was measured using a UV/Vis Nano Spectrophotometer (Quimigen, Madrid, Spain). Subsequent steps included reduction with DTT, alkylation with IAA, enzymatic digestion with trypsin (1:40 ratio), and final peptide recovery after lyophilization and resuspension for LC-MS analysis.

### 2.8. Protein Denaturation, Alkylation, Enrichment, and Digestion

Initially, the samples were standardized to a concentration of 1 mg/mL using milliQ water, followed by reduction with DTT (200 mM, pH 7.8) for a duration of 1 h at 60 °C. Subsequently, IAA (200 mM, pH 7.8) was introduced to facilitate the alkylation of cysteine residues, with an incubation period of 30 min at 37 °C. The digestion of proteins commenced with the addition of trypsin at a ratio of 1:40, and the mixture was incubated for an additional 16 h. The digestion process was terminated by the addition of 5% acetic acid, adjusting the pH to 5. The samples were then subjected to a drying process for 2 h in a vacuum concentrator (lyophilizer Freezone 2.5 freeze dryer Benchtop, Labconco, Kansas City, MO, USA) at a temperature of −40 °C and a vacuum pressure of 0.080 mBar. Following lyophilization, the peptides were eluted in a solution of 0.1% acetic acid and acetonitrile (98:2 *v*/*v*) to achieve a final concentration of 100 µg/µL.

### 2.9. HPLC-MS/MS-QTOF Mass Spectrometry and Data Analysis

#### 2.9.1. Mycotoxins

Chromatographic analysis was performed using an Agilent Technologies 1200 Infinity Series LC system (Santa Clara, CA, USA) coupled with an Agilent 6540 UHD Accurate-Mass HPLC-MS/MS-QTOF, featuring an electrospray ionization system with the Agilent Dual Jet Stream ion source (Dual AJS ESI). Mycotoxin separation was carried out as previously optimized by [18]. A Gemini C18 column (50 mm × 2 mm, 110 Å, 3 μm particle size; Phenomenex, Palo Alto, CA, USA) was used for the analysis. The mobile phases consisted of water (solvent A) and acetonitrile (solvent B), each containing 0.1% formic acid. The elution was carried out at a flow rate of 0.3 mL/min, with the following gradient: 0 min, 5% B; 30 min, 95% B; 35 min, 5% B. The total analysis time was 25 min with a 5 µL injection volume. For mass spectrometry, a 6540 Agilent Ultra High-Definition Accurate Mass MS/MS-QTOF system, coupled to an Agilent Dual Jet Stream electrospray ionization (Dual AJS ESI) interface in positive ion mode, was utilized. The mass spectrometry parameters were optimized as follows: fragment voltage 175 V, capillary voltage 3.5 kV, collision energies of 10, 20, and 40 eV, nebulizer pressure at 30 psi, drying gas flow (N2) set to 8 L/min, and a drying gas temperature of 350 °C. Data analysis was conducted using MassHunter Qualitative Analysis Software B.08.00 (Agilent Technologies, Santa Clara, CA, USA).

#### 2.9.2. Phenolic Compounds

Chromatographic separation was performed as described by [25]. The mobile phases consisted of H_2_O with 1% acetic acid as solvent system A and acetonitrile as solvent system B, with the following gradient elution profile: 0 min, 0.8% B; 5.5 min, 6.8% B; 16 min, 20% B; 20 min, 25% B; 25 min, 35% B; 29 min, 100% B; 32 min, 100% B; 34 min, 0.8% B; 36 min, 0.8% B. The column was equilibrated for 3 min before each analysis. The injected sample volume was 20 μL, and the flow rate was set at 0.8 mL min^−1^. MS analyses were conducted using HPLC-MS/MS-QTOF operating in negative ionization mode. The settings were as follows: drying gas flow (N_2_), 12.0 L min^−1^; nebulizer pressure, 50 psi; gas drying temperature, 370 °C; capillary voltage, 3500 V; fragmentor voltage, 3500 V, and scan range *m*/*z* 50–1500. Automatic MS/MS experiments were performed with collision energy values set at: *m*/*z* 100, 30 eV; *m*/*z* 500, 35 eV; *m*/*z* 1000, 40 eV; and *m*/*z* 1500, 45 eV. Data integration and processing were conducted using Mass Hunter Workstation software, version 10.0 (Agilent Technologies). Mass Hunter Personal Compound Database and Library (PCDL) Manager Software, version B.08.00 (Agilent Technologies) was used for untargeted compound identification.

#### 2.9.3. Carotenoids

The carotenoid profile was analyzed by following the method validated by [26], with slight modifications. The mobile phase consisted of a time-programmed gradient with methanol (containing 0.1% acetic acid) as phase A and a mixture of MTBE and methanol (80:20, *v*/*v*) containing 0.1% acetic acid as phase B. The gradient profile was as follows: equilibration for 2 min at 90% A, followed by a decrease from 90% to 75% A over 10 min, then from 75% to 50% A in another 10 min, from 50% to 30% A over 5 min, 30% to 10% A in 5 min, then 10% to 6% A in 2 min, followed by a return to the initial conditions in 2 min and a 3 min hold. The flow rate remained constant at 0.40 mL/min throughout. The total run time was 50 min. MS analyses were conducted operating in negative ionization mode. For MS/MS analysis we used the following parameters: drying gas flow (N_2_), 8 L min^−1^; nebulizer pressure, 55 psi; gas drying temperature, 350 °C; capillary voltage, 3500 V; fragmentor voltage, 110 V, and scan range *m*/*z* 70–1100. Automatic MS/MS experiments were performed with collision energy values set at 5 *m*/*z*, 45 eV. Data integration and processing were conducted using Mass Hunter Workstation software (Agilent Technologies).

#### 2.9.4. Proteomic Analysis

For proteins analysis, a C18 bioZen™ column (2.6 μm, 120 Å, 50 × 2.1 mm, Phenomenex, CA, USA) was employed to facilitate the chromatographic separation of peptides. The injection volume was set at 10 μL, and the column temperature was maintained at 45 °C. The total analysis time was 40 min, with a flow rate of 0.4 mL/min. Also, in this case the mobile phases consisted of two components: A (H_2_O with 0.1% formic acid) and B (ACN with 0.1% formic acid). A gradient elution was implemented, starting with 3% phase B for the first minute, increasing to 20% over 21 min, then ramping up to 95% in 3 min, and finally returning to 3% over the last 10 min. Regarding the QTOF-MS parameters, the drying gas flow (N_2_) was set to 13.0 L/min, with a nebulizer pressure of 35 psi and a gas drying temperature of 325 °C. The capillary voltage was maintained at 4000 V, the nozzle voltage at 500 V, the fragmentor voltage at 175 V, the skimmer voltage at 65 V, and the octopole RF peak at 750 V. Positive ionization was achieved using a Dual AJS ESI interface, with positive ions detected in the m/z range of 100–3000 for MS scans and 50–3000 for auto MS/MS scans, at scan rates of 8 scans/s for MS and 3 scans/s for MS/MS, respectively. The MS/MS automatic acquisition utilized ramped collision energy with a charge state preference of 2, 3, and >3, with slopes of 3.1, 3.6, and 3.6, respectively. Two reference masses, 121.0509 and 922.0098 *m*/*z*, were employed for internal mass calibration. The Agilent MassHunter Workstation software B.08.00 (Agilent Technologies) served as the instrument controller and for data acquisition.

### 2.10. Bioinformatics and Statistical Analysis

The spectra were analyzed by Spectrum Mill MS Proteomics Workbench Package Rev BI.07.11.216 software from Agilent Technologies, which effectively processes high-quality spectra to minimize false positives and employs the Uniprot catalog for protein and peptide identification. The species corresponding to the UniProt codes used in the bioinformatic analysis was Homo sapiens. The MS/MS parameters of the spectra were examined and validated following the methodology outlined by [27]. Identified proteins were subsequently exported to Mass Profiler Professional (MPP) version 15.0 (Agilent Technologies) for statistical analysis. This analysis focused on the total spectral intensity of proteins treated as entities within MPP. The baseline of the spectra was normalized to the mean across all samples. Entities were filtered based on their occurrence frequency across all replicates of a single group. Comparisons between the experimental mycotoxin dose and the control were conducted using an unpaired *t*-test with Benjamini–Hochberg adjustment. Results were deemed significant with fold change (FC) values of ≥1.5 for upregulated proteins and ≤−1.5 for downregulated proteins, with *p*-value thresholds set at <0.05. Finally, biological processes, molecular functions, and metabolic pathways associated with these proteins were meaningfully identified using the DAVID database for integrated protein annotation, visualization, and discovery [28,29]. Graphical representation of DAVID data and heatmap visualization was performed with GraphPad Prism version 10.1.1 for Macintosh (GraphPad Software, Boston, MA, USA, www.graphpad.com). Metabolic pathway enrichment was carried out through the Reactome Database [30] and STRING tool for the retrieval of interacting proteins [31]. Protein–protein interaction (PPI) networks were built using the STRING database. Interaction networks were integrated by ConsensusPathDB [32].

## 3. Results and Discussion

### 3.1. Intestinal Digest Profile

#### 3.1.1. Mycotoxin Concentration

To initiate the in vitro exposure of SH-SY5Y cells, the first step was to quantify the mycotoxin levels in the intestinal digests of bread with functional ingredients through an HPLC-MS/MS-QTOF system. The determination of mycotoxin quantity in the digest was crucial for adjusting it to a dilution that best matched the 100 nM concentration set for the control, ensuring that it remained below cytotoxicity levels for the cells. The experimental conditions and the concentrations detected in the diluted digest (1:50) are shown in Table 2. As can be seen, the levels of OTA (141 to 207 nM) were consistently higher than that of AFB1 (28 to 39 nM), both in the bread with just pumpkin and in the one containing both ingredients. To explain this difference in concentration between the two mycotoxins, it is important to emphasize that AFB1 has been shown to be sensitive to typical bread-making temperatures (usually between 180 and 200 °C) [33]. Moreover, this was confirmed by the fact that no significant decrease in AFB1 levels was observed during baking processes, even at higher temperatures used in the thermal treatment of other foods [34]. As for OTA, evidence suggests that it remains highly stable throughout the breadmaking process, with no significant reduction in its content during kneading, fermentation, or baking [35]. Furthermore, considering the bioaccessibility aspect after simulated in vitro digestion, findings revealed significant percentages of reduction using functional ingredients [36,37]. In particular, when employing FW + P as bioactive components, the reductions reached 68% for AFB1 and 11% for OTA, with AFB1 degradation still being higher than OTA [18].

#### 3.1.2. Phenolic Profile

The phenolic profile of the digested extract of bread used in this work was obtained through an untargeted HPLC-MS/MS-QTOF analysis. Table S1 shows the main compounds detected (score > 80) acquired in negative mode. All the spectra are included in the Supplementary Material S2. They include phenolic acids: *p*-coumaric acid, sinapic acid, caffeic acid, ferulic acid, vanillic acid, benzoic acid; hydroxycinnamic acids: 3-4-dihydroxyhydrocinnamic acid and hydroxycinnamic acid; other compounds such as vanillin, 1-2-dihydroxybenzene, lactic acid and DL-3-phenyllactic acid, which is well known for being a biosafe antimicrobial compound mainly produced by lactic acid bacteria, known for its wide-ranging antimicrobial activity [38]. The interest in using these functional ingredients lies in the fact that these compounds have been widely studied for their strong antioxidant and anti-inflammatory capabilities. In fact, they have been reported to prevent oxidative damage diseases and contribute to overall health improvement [39]. Furthermore, it is important to note that these compounds are stable or minimally affected (or sometimes increased) by the various processing treatments [40].

#### 3.1.3. Carotenoids

The extract of the intestinal digest was also analyzed for its carotenoid profile, including samples exposed to PID and PID + FW, both in the presence and absence of mycotoxins. Table S2 shows the average concentration and profile of major carotenoids identified in digested bread extracts, more specifically β-carotene, lutein, antheraxanthin, violaxanthin, z-zeaxanthin and β-cryptoxanthin. In general, the carotenoid content was higher in the extract combined with the FW than in the one from the bread with only the addition of pumpkin. In both cases (PID or PID + FW, respectively), the highest concentrations were recorded for β-carotene (0.2 to 0.7 µg/mL–1.8 to 4 µg/mL), violaxanthin (0.49 to 1.33 µg/mL–2.7 to 3.39 µg/mL), and β-cryptoxanthin (0.36 to 1.02 µg/mL–1.8 to 2.9 µg/mL).

### 3.2. Cell Viability

**Figure 2:**
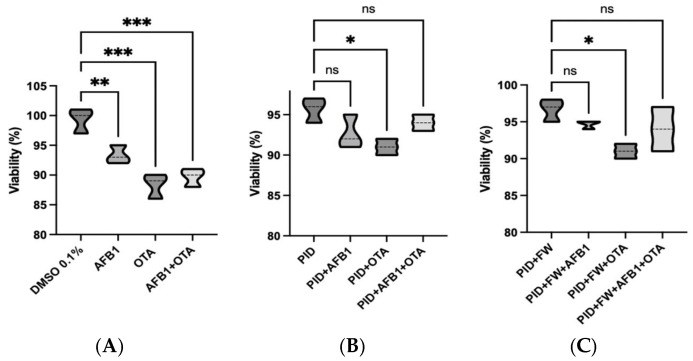
Violin plots showing cell viability of differentiating SH-SY5Y cells after 7 days of exposure to mycotoxin standard in organic solvent (DMSO) (**A**), diluted intestinal digest of bread with pumpkin (PID) (**B**), diluted intestinal digest of bread with pumpkin and fermented whey (PID + FW) (**C**). * *p* < 0.05, ** *p* < 0.01, *** *p* < 0.001 significantly different from the control.

A cell viability assay was carried out on SH-SY5Y cells undergoing differentiation after 7 days of exposure to standard mycotoxins and digested bread extracts in order to confirm the absence of cytotoxicity, using concentrations lower than the IC_50_ (Figure 2). As demonstrated by previous studies conducted with the same cell line, IC_50_ values of 100 µM for AFB1 and between 5.80 and 50 µM for OTA have been reported [41]. In this study, nearly the 90% viability was observed for all conditions analyzed. However, it should be noted that both the OTA standard (Figure 2A) and OTA in digests (Figure 2B) induces a significant reduction in viability compared to the control. Indeed, the effect of OTA appears to be more pronounced than that of AFB1 and the mycotoxin mixture.

Notably, when comparing each mycotoxin exposure to its corresponding co-treatment with functional ingredients (as shown in Figure S1), no significant differences in cell viability were observed. However, a trend toward improved viability was consistently observed in the co-treated groups. This suggests a potential protective effect of the bioactive compounds, which, while not statistically significant under the present conditions, may contribute to maintaining cellular homeostasis and mitigating early toxic insults. Importantly, cell viability remained high (>85%) across all co-treated samples, further supporting the safety and biological relevance of the applied treatment conditions.

In addition to cell viability, the potential of these bioactive ingredients to counteract AFB1- and OTA-induced neurotoxicity has already been demonstrated in our previous study, where the same extracts were shown to alleviate toxin-induced alterations in neuronal differentiation and promote the expression of neuronal markers in SH-SY5Y cells. This provides additional support for the biological relevance of the tested concentrations and treatment conditions [5].

### 3.3. Identification and Quantification of Differentially Expressed Proteins (DEPs)

**Figure 3:**
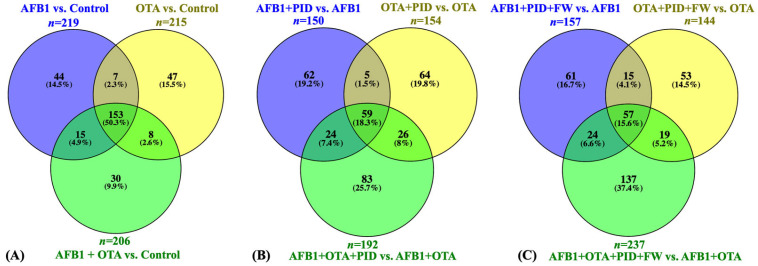
Venn diagram representation of common DEPs for SH-SY5Y cells exposed to mycotoxins versus the control (**A**), mycotoxins diluted in intestinal digest of bread with pumpkin (PID) (**B**), or PID with fermented whey (PID + FW) (**C**) versus the corresponding mycotoxin group. *p* < 0.05 were significantly different from the control.

To further investigate the impact of these ingredients on protein expression, various statistical analyses were performed to clarify their mechanism of action in the presence of toxins and functional additives. The results of the statistical comparison between cells exposed solely to mycotoxins and the control revealed a total of 219 DEPs for AFB1, 215 for OTA, and 206 for the combination of AFB1 and OTA, with 153 common proteins across the three treatments (Figure 3A). However, in the presence of intestinal digest, the number of significant proteins was reduced. When the intestinal digest from bread and pumpkin was used, 150 proteins were identified for AFB1, 154 for OTA, and 192 for the combination of both mycotoxins (Figure 3B). The addition of FW further modified the protein profile, with 157 proteins for AFB1, 144 for OTA, and 237 for the mycotoxin mixture. Specifically, 59 proteins were common for the treatment with bread and PID, while 57 were common for the treatment with PID and FW (Figure 3C). Given the known toxicity of these toxins, attention is directed toward the decrease in the number of proteins when functional ingredients are introduced.

Considering the overall expression of DEPs, Figure S2 shows how their regulation (LogFC) varies with respect to each condition: mycotoxins only (A), mycotoxins + PID (B), and mycotoxins + PID + FW (C). Starting with AFB1, it induced a gradual widening of the distribution, suggesting a heterogeneous response across samples, with significant variation in protein expression from negative to positive values. However, a decrease of proteins down to LogFC = −6 was observed, but with a greater prevalence of the proteins that are strongly upregulated (LogFC = 13), exhibiting different variability. The addition of the digest (B) showed a more irregular distribution of SH-SY5Y responses, with higher expansion of the LogFC, indicating its modulation compared to AFB1 alone. In fact, the upregulated proteins were maintained, but a portion of them also showed strong downregulation (LogFC = −18). The situation remains similar with both ingredients, but with a slight reduction in repression (LogFC = −9) and reduction in variability and extreme responses. OTA showed a narrower pattern, but still widely distributed, with more pronounced effects in the negative region and a moderate positive response. As observed, by adding the functional ingredients, a marked decrease is evident down to LogFC = −18, with reduced values of upregulated proteins, especially when both ingredients were present (LogFC up to 10).

Similar to other studies, a more interesting scenario is observed with both mycotoxins. Regarding this, previous findings have demonstrated a combined interaction of AFB1 and OTA, contributing to the suppression of metabolism, immune function, and antioxidant status [42]. Similarly, AFB1 and OTA were more cytotoxic in combination than alone, forming an additive combination [43]. These findings indicate that the AFB1 and OTA mixture produces a combined protein response with fluctuations in LogFC values, suggesting that the blend of the two mycotoxins amplifies variability in the cellular response. The inclusion of P in the formulation exerts once again a modulatory influence, reducing the intensity of the more extreme downregulated protein responses (LogFC down to −3), even though it increases the upregulated proteins (LogFC up to 12). Nevertheless, even considering the impact of the two mycotoxins together, the addition of P and FW reduced the overall variability in logFC values (ranging from −7 to 10), indicating a protective role.

### 3.4. Gene Ontology of Differentially Expressed Proteins

**Figure 4:**
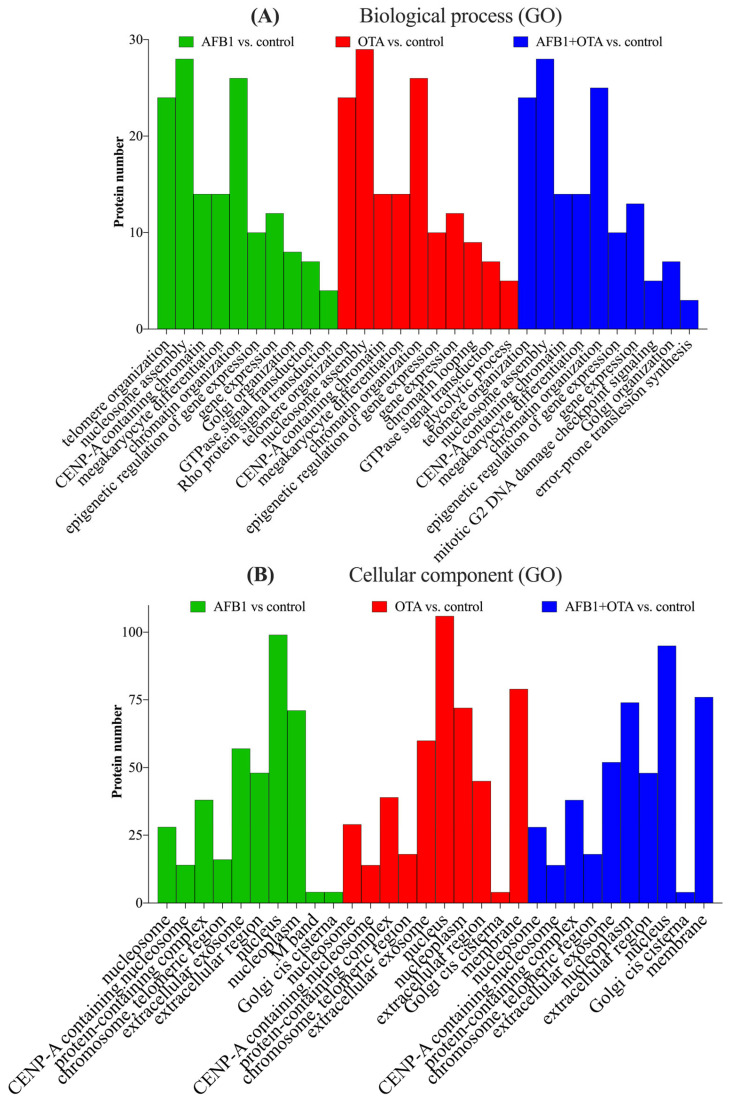
Gene ontology (GO) functional annotation of differentially expressed proteins for biological processes (BP) (**A**) and cellular components (CC) (**B**) of cells exposed to AFB1, OTA, AFB1 + OTA compared with the control.

The analysis of SH-SY5Y neuronal cells exposed to AFB1 and OTA leads to interesting implications for neurotoxicity and the understanding of how these mycotoxins can influence crucial molecular processes in neuronal cells. SH-SY5Y cells, which are a human cell line derived from neuroblastoma, are widely used as a model to study neurodegeneration, oxidative stress, and other cellular alterations in neurons [44]. Gene ontology (GO) functional analysis of the biological processes (BPs) involved suggests that AFB1 and OTA affected key cellular processes including telomere organization (*n* > 20), nucleosome assembly (*n* = 30), and protein localization to CENP-A containing chromatin (*n* > 10) (Figure 4A). These processes are closely interconnected with each other in the maintenance of chromatin integrity, genome regulation, as well as in cell division and DNA stability [45]. In line with this, chromatin organization was also a pivotal process, with a considerable number of proteins involved (*n* > 25). This is significant, as telomerase activity is abnormally high in cancer cells, allowing them to maintain telomere length and counteracting the normal shortening process that occurs during cell division [46]. Indeed, other studies using the same cell line have reported that abnormal telomere elongation was linked to a higher frequency of micronuclei, nucleoplasm bridges, and nuclear buds [47]. Moreover, CENP-A nucleosomes have been associated, in several investigations, with defects in human cells [48,49], which, in this case, were exacerbated by the presence of mycotoxins.

The biological processes mentioned have been shown to be tightly associated with specific cellular components (CCs) that are essential for DNA stability, cell division, and the maintenance of cellular integrity. (Figure 4B). These processes interact with components such as the nucleosome for gene expression regulation and DNA protection, CENP-A for cell division and genomic stability, telomeres for the protection and maintenance of DNA integrity, and the nucleoplasm and Golgi apparatus for the transport of proteins involved in these functions. It is important to emphasize that alterations in these processes due to AFB1 and OTA, either individually or in combination, can compromise cellular function and genetic stability, and have negative impacts on crucial biological processes such as cell division, DNA repair, and neuronal proliferation [50,51].

**Figure 5:**
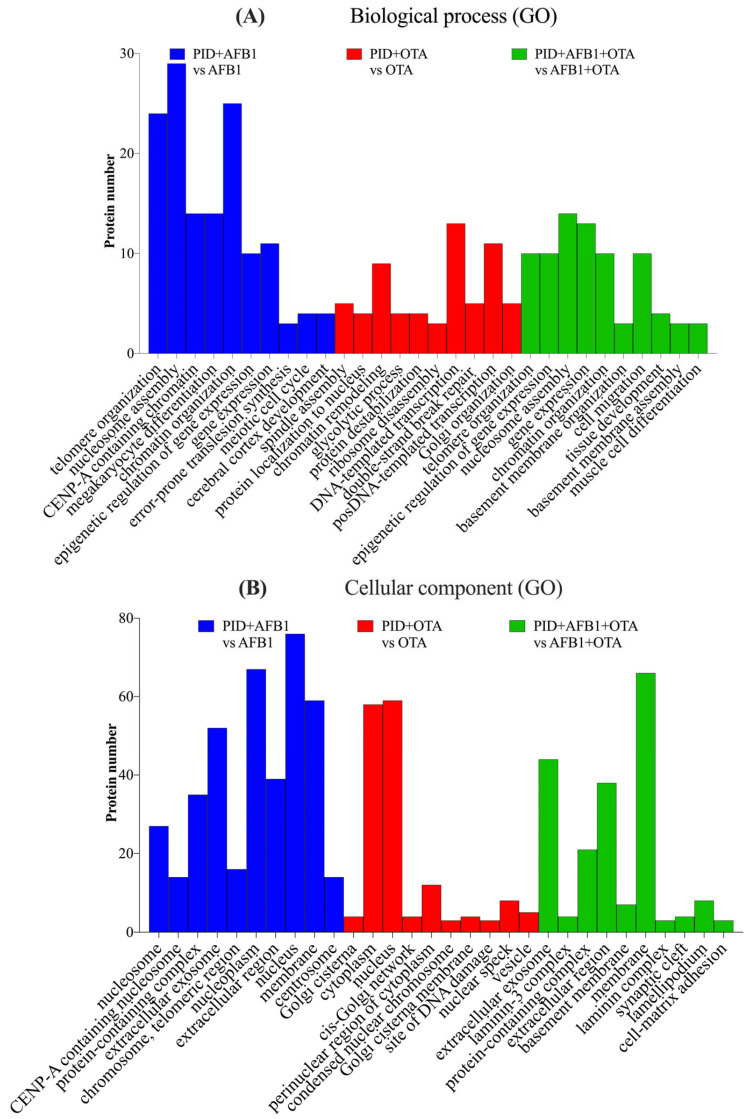
Gene ontology (GO) functional annotation of differentially expressed proteins for biological processes (BP) (**A**) and cellular components (CC) (**B**) of cells exposed to PID + AFB1, PID + OTA, PID + AFB1 + OTA compared with respective mycotoxins without functional ingredient.

When the cells were exposed to AFB1, OTA, or their combination with PID, BPs and CCs similar to those observed with the mycotoxins alone were identified, but with a significantly reduced number of proteins, suggesting a mitigation of the toxic effects. For AFB1, telomere organization, nucleosome assembly, and protein localization to CENP-A-containing chromatin were the most significant processes (Figure 5A), with the nucleosome and CENP-A nucleosome being the prominent CCs (Figure 5B). A similar pattern was observed with both mycotoxins, though the number of proteins was greatly reduced (fewer than 10). In the case of OTA, spindle assembly, positive regulation of protein localization to the nucleus, and chromatin remodeling emerged as the most significant processes, with fewer than 10 proteins involved. Cellular components such as the cisternae of the Golgi, the cytoplasm, and the nucleus were significant for OTA, while for both toxins, the extracellular exosome, laminin-3 complexes, and protein-containing complexes were identified as the most significant.

**Figure 6:**
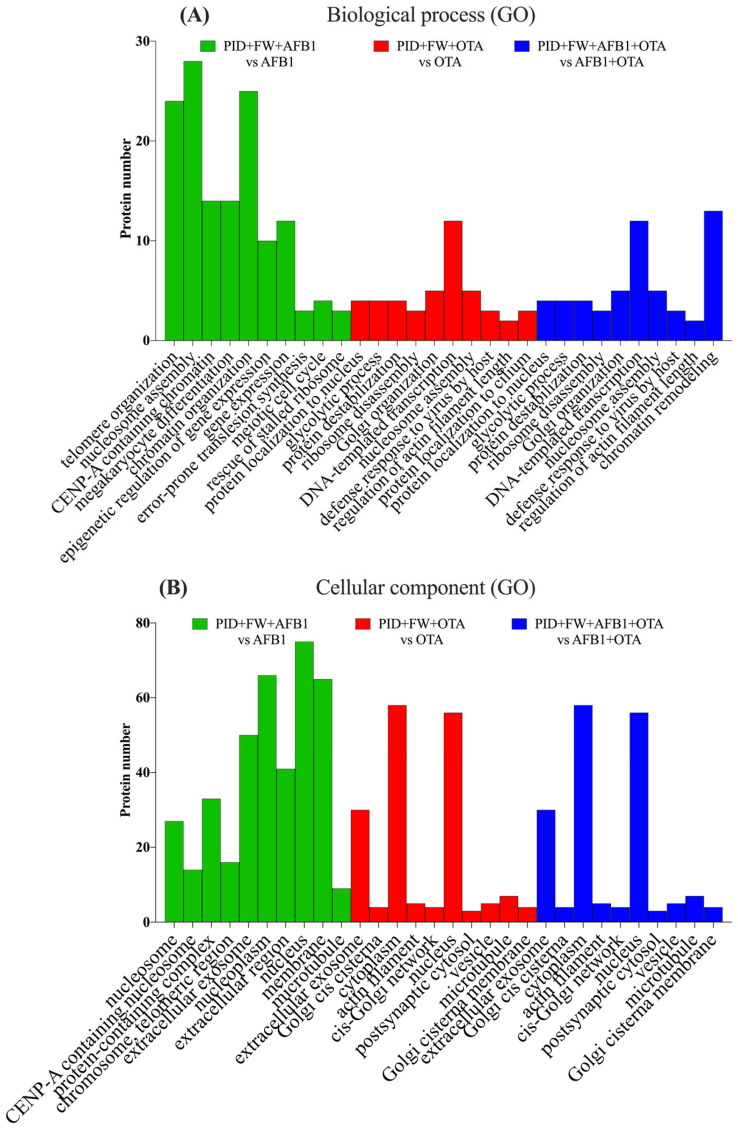
Gene ontology (GO) functional annotation of differentially expressed proteins for biological processes (BP) (**A**) and cellular components (CC) (**B**) of cells exposed to PID + FW + AFB1, PID + FW + OTA, PID + FW + AFB1 + OTA compared with respective mycotoxins without functional ingredient.

When the digestive extract of bread prepared with P and FW was present, the trend observed for AFB1 with PID individually was noted, with a concordant result for both BPs (Figure 6A) and CCs (Figure 6B). For OTA, positive regulation of protein localization to the nucleus, glycolysis, and protein destabilization emerged as the most significant processes, in a manner identical to AFB1 + OTA, but with fewer than five proteins involved.

**Figure 7:**
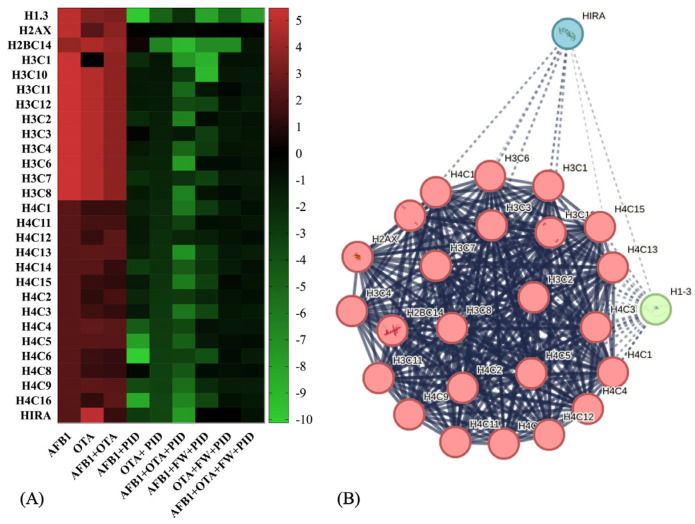
Heatmap representation of protein expression for histone-related activity after exposure to different conditions (**A**) and STRING protein–protein interaction (PPI) network (**B**). Red-to-green gradient represents logarithmic fold change value for upregulated (logFC > 0) and downregulated (logFC < 0) features. Black box is logFC = 0. *p* < 0.05 significantly different from control or mycotoxin groups. For PPI visualization, colored nodes represent clusters and edges represent protein–protein associations.

Considering the common features across all exposures and related to the most important BPs, both with and without functional ingredients, Figure 7A graphically illustrates how the trend of their expression varies. As observed, primarily proteins from the histone family were involved, being strongly upregulated in the presence of AFB1 and OTA. In fact, among the altered ones are mainly those belonging to the H3-H4 tetramer and H2A-H2B dimer, which are integral components of the nucleosome structure, responsible for chromatin organization and regulating various DNA-related processes, including transcription, replication, and repair [52]. This pattern of histone acetylation is critical for neuronal differentiation and neural development, as the proper regulation of gene expression is essential for the maturation of nerve cells [53]. In SH-SY5Y cells, for instance, it has been reported that natural bioactive ingredients play an essential role in modifying cell differentiation [54].

The network visualization analysis (Figure 7B) represents a predicted set of interactions among histone proteins, highlighting functional connections between different variants and their regulators. The central cluster (red nodes), predominantly composed of H3, H4, and H2AX histones, exhibits high internal connectivity, showing a key stable core in chromatin structure and dynamics. On the other hand, HIRA (blue) and H1-8 (green) nodes belong to a broader set that includes chromatin remodeling factors and histone regulators that interact more dynamically to influence chromatin function.

After exposure to mycotoxins, histones and their variants showed a significant increase (up to logFC = 5), with a less pronounced change observed for the histone H4 family (logFC ranging from 0.2332 to 5). Additionally, the histone cell cycle regulator (HIRA) protein, which is involved in the deposition of histone variants and chromatin remodeling [55], is upregulated particularly in the presence of OTA (LogFC = 5). This upregulation of HIRA indicates a potential mechanism for responding to DNA damage or chromatin remodeling triggered by mycotoxin exposure. In all cases, with the addition of functional ingredients, this activity was reduced, decreasing the excessive proliferation induced by tumor cells and exacerbated by mycotoxins, showing fold change expressions ranging from 0 to −8 (Figure 7A). Predicted interactions centered around HIRA as the central node were generated using ConsensusPathDB (http://consensuspathdb.org, accessed on 3 March 2025) (Figure S3). As observed, HIRA is directly correlated with histones H3F3A, HIST2H3A, and HIST1H2BK, emphasizing its function in histone deposition and nucleosome assembly [56], further supported by its link with Anti-Silencing Function 1A Histone Chaperone (ASF1A) [57]. On the other hand, interactions with Histone acetyltransferase p300 (EP300), CCCTC-binding factor (CTCF), and methyl-CpG binding protein 2 (MECP2) indicate its role in chromatin modification and epigenetic regulation, closely related to brain functions [58,59,60]. Additionally, an important function for neural growth is related to the Wnt/β-catenin signaling pathway, which includes transcription factor 7-like 2 (TCF7L2) [61]. Furthermore, HIRA interacts with phosphorylated Cyclin-Dependent Kinase 2 (p-T160-CDK2), pointing to its involvement in cell cycle progression and brain development [62]. Lastly, its association with RNA polymerase activity and transcriptional regulation is demonstrated by its interaction with transcription factors and other co-activators that regulate gene expression, such as RNA polymerase II subunit A (POLR2A), Basic Transcription Factor 2 (BRF2), BRF1-Dependent RNA Polymerase II Transcriptional Factor (BDP1), and General Transcription Factor IIIC Subunit 1 (GTF3C1) [63,64,65,66].

### 3.5. Metabolic Pathways Analysis and Enrichment

Upon further examination of the metabolic pathways influenced by the action of DEPs in comparison to the control, a Reactome evaluation and visualization was performed to define the most relevant pathways. In Figure S4, the metabolic pathways identified for AFB1 (A), OTA (B), and the combination of both mycotoxins (C) are displayed. As observed, in the presence of all three conditions, the most significant and enriched process appears to be associated with the RHO GTPases family, with a protein count similar across all conditions (*n* = 25). However, in the presence of OTA, signal transduction is also observed as the most significant, with a high number of proteins involved (*n* = 45). Among others, the cellular stress response and the cell cycle emerge as key pathways after AFB1 and the mixture exposure (Figure S4A–C).

Interestingly, estrogen-dependent gene expression (*n* < 10), androgen receptor transcription stimulated by protein kinase C-related kinase 1 (PKN1) activation (*n* < 5), and histone acetyltransferases (HATs) (*n* = 7) stood out. According to that, it has been demonstrated that diverse estrogen receptors, such as estrogen receptor alpha (ERα), can regulate the malignant invasion process of neuroblastoma, being positively expressed when stimulated by xenobiotics [67]. Concretely, several studies have demonstrated the regulatory relationship between the estrogen receptor and SH-SY5Y cells [68,69,70]; furthermore, AFB1 and OTA can influence estrogenic activity, potentially modulating the expression of estrogen receptors [71,72,73]. On the other hand, PKN1 is the predominant isoform of the serine/threonine protein kinase C-related kinase (PKN/PRK) family in neurons, playing a crucial role in various functions such as cytoskeletal organization and neuronal differentiation. Its dysregulation has been linked to neuropathological conditions like amyotrophic lateral sclerosis and Alzheimer’s disease [74].

In the presence of the digestive extract of bread with pumpkin (Figure S5), RHO GTPase activity is once again observed, but in a distinct manner from the conditions with mycotoxins alone, being more pronounced with AFB1 (*n* = 20), followed by OTA (*n* = 17), and lastly by the combination of AFB1 and OTA (*n* = 10). On the other hand, the cell cycle appears to be the most significant process in the presence of AFB1 (*n* = 19), while protein metabolism (*n* = 41) and signal transduction (*n* = 40) are most prominent with the combination of AFB1 and OTA. Interestingly, a similar trend is observed with both functional ingredients (Figure S6). Specifically, RHO GTPases maintain a comparable pattern, though again slightly lower with OTA (*n* = 16) compared to AFB1 (*n* = 19) and the combination of both (*n* = 19). In the case of the AFB1 + OTA mixture, the disease pathway emerges as a prominent metabolic pathway (*n* = 24). Additionally, several other processes stand out, including the cellular stress response, NORC (Nuclear Receptor Corepressor), B-WICH complex, cellular senescence, and androgen receptor activity (notably in AFB1 and the combination).

**Figure 8:**
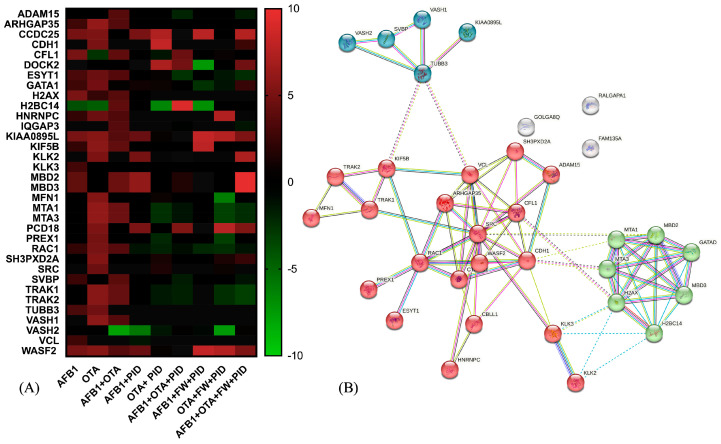
Heatmap representation of protein expression for Rho GTPase-related activity after exposure to different conditions (**A**) and the STRING protein–protein interaction (PPI) network (**B**). The red-to-green gradient represents the logarithmic fold change value for upregulated (logFC > 0) and downregulated (logFC < 0) features. For PPI visualization, colored nodes represent the clusters and edges represent the protein–protein associations.

Deepening the analysis of pathway enrichment, the heatmap visualization (Figure 8A) reports the expression of proteins belonging to the Rho GTPase cycle in response to the different treatments. As observed, several proteins show increased expression (red) with AFB1 and OTA, suggesting a cellular stress response. Interestingly, when adding functional ingredients (PID, FW), many proteins that were overexpressed under mycotoxin exposure return to baseline levels (black or green), suggesting a regulatory or protective effect. On the other hand, Figure 8B highlights a key regulatory module centered around RHO GTPase signaling through the visualization of a predicted PPI network. As observed, three significant clusters are interconnected, underscoring cytoskeletal dynamic and signal transduction (red cluster), chromatin remodeling (green) and microtubule associated proteins (blue). This complex interplay of interactions could have significant implications in cancer metastasis, neurological diseases, and cellular homeostasis.

Considering the red cluster, key regulators such as Ras-related C3 botulinum toxin substrate 1(RAC1), Wiskott-Aldrich Syndrome Protein Family Member 2 (WASF2), and Rho GTPase Activating Protein 35 (ARHGAP35) govern cytoskeletal remodeling by modulating actin polymerization and depolymerization dynamics. These interactions are crucial for processes such as cell migration, invasion, and adhesion, which are often dysregulated in cancer metastasis. Going further, the loss or inactivation of the tumor suppressor ARHGAP35 has been associated with cancer progression and neurodevelopmental pathways [75], as in the case of AFB1 in SH-SY5Y cells (LogFC = −2.31). Interestingly, it was upregulated with AFB1 + PID + FW (LogFC= 2.72). Additionally, ARHGAP35 expression was also increased in the AFB1 + OTA condition (LogFC = 3.604) when compared to the control and was further upregulated with the addition of PID (LogFC = 11.48), suggesting a potential synergistic effect. The presence of proto-oncogene SRC further suggests a link between Rho GTPase signaling and tyrosine kinase-mediated pathways, reinforcing the role of this module in tumor progression and cellular signaling.

The green cluster, enriched in chromatin remodelers, indicates a potential crosstalk between cytoskeletal regulation and epigenetic modifications. Among these, there are metastasis-associated proteins 1 and 3 (MTA1, MTA3), and methyl-CpG binding domain protein 2 and 3 (MBD2, MBD3), which interact as part of the nucleosome remodeling and histone deacetylation (NuRD) multi-subunit protein complex, regulating in turn cellular differentiation as well as cell fate transitions in a variety of cell types and developmental contexts [76,77]. In SH-SY5Y cells, MTA1 and MTA3 levels were significantly elevated after OTA (LogFC > 6) and mycotoxin mixture (LogFC > 4) exposure, as demonstrated by [78], where high levels of these proteins led to cerebral ischemia. However, MTA1 may have an opposing effect by influencing nNOS-induced oxidative stress or modulating ERα signaling-related apoptotic pathways in a detrimental manner [79]. When the functional ingredients were added, the expression was completely reversed, with LogFC values ranging from −1.23 to 3.23.

Lastly, the blue cluster consists of diverse microtubule-associated proteins, essential for multiple cellular functions such as the regulation of neuronal differentiation, cytoskeletal organization, intracellular transport, and mitotic spindle formation, all of which are crucial for proper neurodevelopment and cellular homeostasis. Notably, Vasohibin (VASH)/small vasohibin binding protein (SVBP) complexes play a key role in neuron polarization and brain cortex development, indicating their involvement in shaping neuronal architecture and ensuring proper cortical formation [80]. In this study, proteins belonging to this complex were altered. More specifically, according to [81], a recovery mechanism that leads to the inhibition of VASH1/VASH2 was triggered after treatment with AFB1, OTA and the AFB1 + OTA mix, becoming more pronounced in the presence of functional ingredients (LogFC down to −7.50). Likewise, SVBP was altered in a similar manner to the VASH1/2 proteins, whose expression was modified by the addition of functional ingredients.

## 4. Conclusions

This investigation demonstrated the beneficial role of FW and P as functional ingredients by clarifying the mechanisms that lead to improvements in responses to neuronal damage exacerbated by mycotoxins at the proteomic level. The intriguing results revealed a connection between proteins primarily associated with nucleosome structure, including chromatin remodeling and histone regulators. Moreover, major controllers involved in the RHO GTPase family were activated in the presence of bioactive compounds. Overall, this study provides very promising support for the use of functional ingredients rich in antioxidant components to reduce the effects of xenobiotics that naturally contaminate food worldwide. Furthermore, it aims to demonstrate the potential of using cell lines to reduce the use of laboratory animals in experimentation.

However, further studies are needed to validate these findings through orthogonal methods such as Western blot or qPCR, which would help further confirm the observed expression variations and strengthen the mechanistic understanding of the effects. These targeted validation experiments will be crucial for enhancing the translational relevance of the proteomic dataset and for further investigating the potential applications of these bioactive ingredients in neuroprotection.

## Figures and Tables

**Table 1:** Bread conditions prepared in the present study. FW concentration (1%), P concentration (1%).

Bread Type
**Pumpkin (P) bread**
Bread + P
Bread + P + AFB1
Bread + P + OTA
Bread + P + AFB1 + OTA
**Fermented whey (FW)-Pumpkin (P) bread**
Bread + FW + P
Bread + FW + P+AFB1
Bread + FW + P+OTA
Bread + FW + P+AFB1 + OTA

**Table 2:** AFB1 and OTA concentrations of each condition employed: mycotoxin standard in organic solvent (DMSO), diluted intestinal digest of bread with pumpkin (PID), diluted intestinal digest of bread with pumpkin and whey fermented (PID + FW). A dilution of 1:50 was used. Data are presented as mean ± standard deviation of technical triplicates.

		Mycotoxin Concentration (nM)	
	Condition	AFB1	OTA
(I)	Control (DMSO ^1^ 0.1%)	-	-
	AFB1	100	-
	OTA	-	100
	AFB1 + OTA	100	100
(II)	Control (PID ^2^)	-	-
	PID +AFB1	28 ± 0.93	-
	PID + OTA	-	156 ± 0.09
	PID + AFB1 + OTA	38 ± 0.45	141 ± 1.02
(III)	Control (PID + FW ^3^)	-	-
	PID + FW + AFB1	39 ± 0.03	-
	PID + FW + OTA	-	207 ± 0.32
	PID + FW + AFB1 + OTA	38 ± 0.12	167 ± 0.05

^1^ DMSO, dimethyl sulfoxide; ^2^ PID, pumpkin intestinal digest; ^3^ FW, fermented whey.

## Data Availability

The original contributions presented in this study are included in the article and Supplementary Materials. Further inquiries can be directed to the corresponding author.

## Supplementary Materials

The following supporting information can be downloaded at https://www.mdpi.com/article/10.3390/antiox14050571/s1, Supplementary Material S1: Figure S1: Violin plots showing cell viability of differentiating SH-SY5Y cells after 7 days of exposure to standard AFB1(A), OTA(B) and AFB1 + OTA (C) compared to corresponding digested bread extracts (PID or PID + FW). PID, digest of bread with pumpkin; PID + FW, digest of bread with pumpkin and fermented whey; ns, not statistically significant. Figure S2: Violin plot displaying the distribution of DEPs for SH-SY5Y cells exposed to mycotoxins versus the control (A), mycotoxins diluted in intestinal digest of bread with pumpkin (PID) (B) or PID with fermented whey (PID + FW) (C) versus the corresponding mycotoxin group. Figure S3: Visualization of HIRA protein interactions generated by ConsensusPath DB software. Figure S4: Functional Reactome pathway enrichment analysis of protein sets across different experimental conditions: (A) AFB1, (B) OTA, (C) AFB1 + OTA. Figure S5: Functional Reactome pathway enrichment analysis of protein sets across different experimental conditions: (A) PID + AFB1, (B) PID + OTA, (C) PID + AFB1 + OTA. Figure S6: Functional Reactome pathway enrichment analysis of protein sets across different experimental conditions: (A) PID + FW + AFB1, (B) PID + FW + OTA, (C) PID + FW + AFB1 + OTA. Table S1: Phenolic compounds identified in intestinal digest of bread enriched with PID + FW by LC-MS/MS-QTOF. Table S2: Concentration (μg/mL) and profile of carotenoids identified from intestinal digests of pumpkin (PID) and PID with fermented whey (FW). Supplementary Material S2: HPLC-MS/MS-QTOF spectra of phenolic compounds identified in the studied extracts.

## Abbreviations

The following abbreviations are used in this manuscript:

ACN	Acetonitrile
AFB1	Aflatoxin B1
ARHGAP35	Activating Protein 35
ASF1A	Anti-Silencing Function 1A Histone Chaperone
BDP1	BRF1-Dependent RNA Polymerase II Transcriptional Factor
BHT	Butylated hydroxytoluene
BP	Biological Process
BRF2	Methyl-CpG binding protein 2
CC	Cellular Component
CTCF	CCCTC-binding factor
DEPs	Differentially expressed proteins
DMSO	Dimethyl sulfoxide
DTT	DL-Dithiothreitol
EP300	Histone acetyltransferase p300
EP300	Histone acetyltransferase p300
ERα	Estrogen receptor alpha
FBS	Fetal bovine serum
FC	Fold Change
FW	Fermented whey
GO	Gene Ontology
GTF3C1	General Transcription Factor IIIC Subunit 1
HATs	Acetyltransferases
HIRA	Histone cell cycle regulator
LAB	Lactic acid bacteria
MBD2	Methyl-CpG binding domain protein 2
MBD3	Methyl-CpG binding domain protein 3
MECP2	Methyl-CpG binding protein 2
MTA1	Metastasis-associated proteins 1
MTA3	Metastasis-associated proteins 3
MTBE	Methyl Tert-Butyl Ether
NORC	Nuclear Receptor Corepressor
OTA	Ochratoxin A
P	Pumpkin
p-T160-CDK2	Phosphorylated Cyclin-Dependent Kinase 2
PBS	Phosphate buffer saline
PID	Pumpkin intestinal digest
PKN/PRK	Serine/threonine protein kinase C-related kinase
PKN1	Protein kinase C-related kinase 1
POLR2A	RNA polymerase II subunit A
PPI	Protein–protein interactions
PTFE	Polytetrafluoroethylene
QTOF	Quadrupole Time-of-flight
RAC1	Ras-related C3 botulinum toxin substrate 1
RHO GTPases	Ras homolog Guanosine Triphosphate
SH-SY5Y	Human neuroblastoma cell line
SVBP	Small vasohibin binding protein
TCF7L2	Transcription factor 7-like 2
VASH	Vasohibin
WASF2	Wiskott-Aldrich Syndrome Protein Family Member 2
