# Supplementary material for: Deepening the Modulatory Activity of Bioactive Compounds Against AFB1- and OTA-Induced Neuronal Toxicity Through a Proteomic Approach
Source: Antioxidants (Basel). 2025 May 9;14(5):571. doi: 10.3390/antiox14050571 (PMC12108279; doi:10.3390/antiox14050571)
Supplement: Supplementary file 1 [file antioxidants-14-00571-s001.zip › Supplementary material S1.pdf]

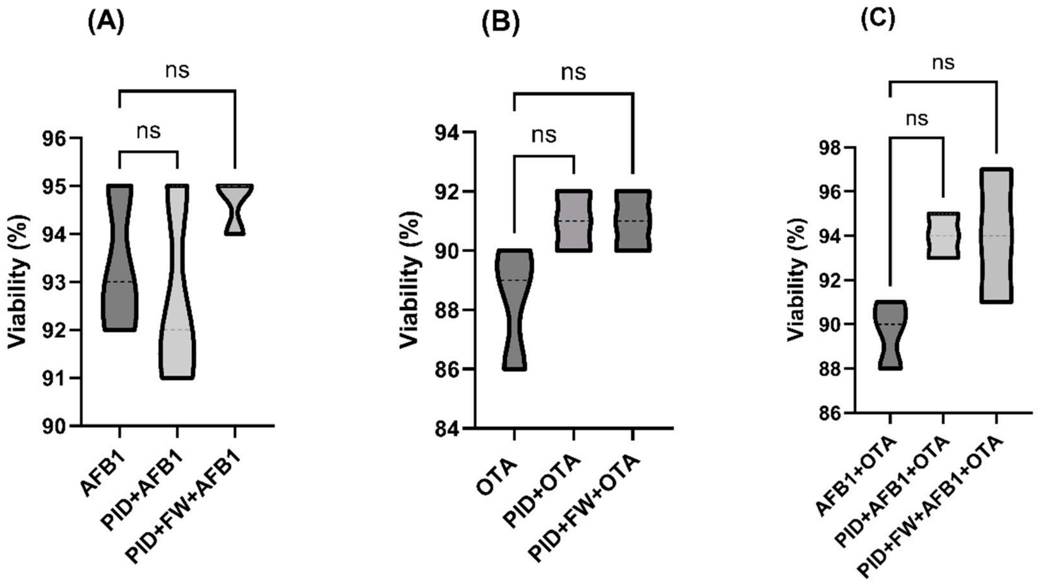

Figure S1. Violin plots showing cell viability of differentiating SH-SY5Y cells after 7 days of exposure to standard AFB1 (A), OTA (B) and AFB1+OTA (C) compared to corresponding digested bread extracts (PID or PID+FW). PID, digest of bread with pumpkin; PID+FW, digest of bread with pumpkin and fermented whey; ns, not statistically significant.

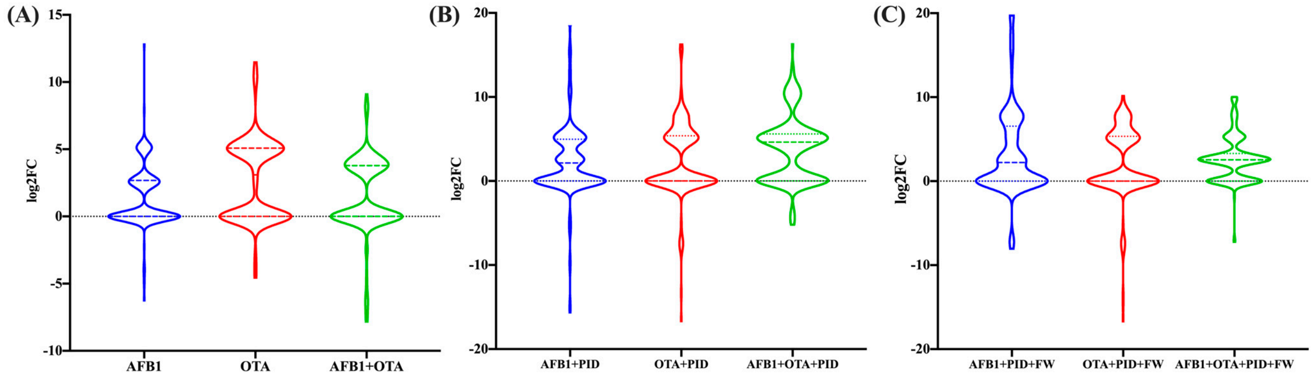

Figure S2. Violin plot displaying the distribution of DEPs for SH-SY5Y cells exposed to mycotoxins versus the control (A), mycotoxins diluted in intestinal digest of bread with pumpkin (PID) (B) or PID with fermented whey (PID+FW) (C) versus the corresponding mycotoxin group. The Y-axis represents the relative expression level of upregulated (LogFC > 0) and downregulated (Log2FC < 0) proteins. All DEPs are proteins that show a p value < 0.05 significantly different from the control or mycotoxin groups.

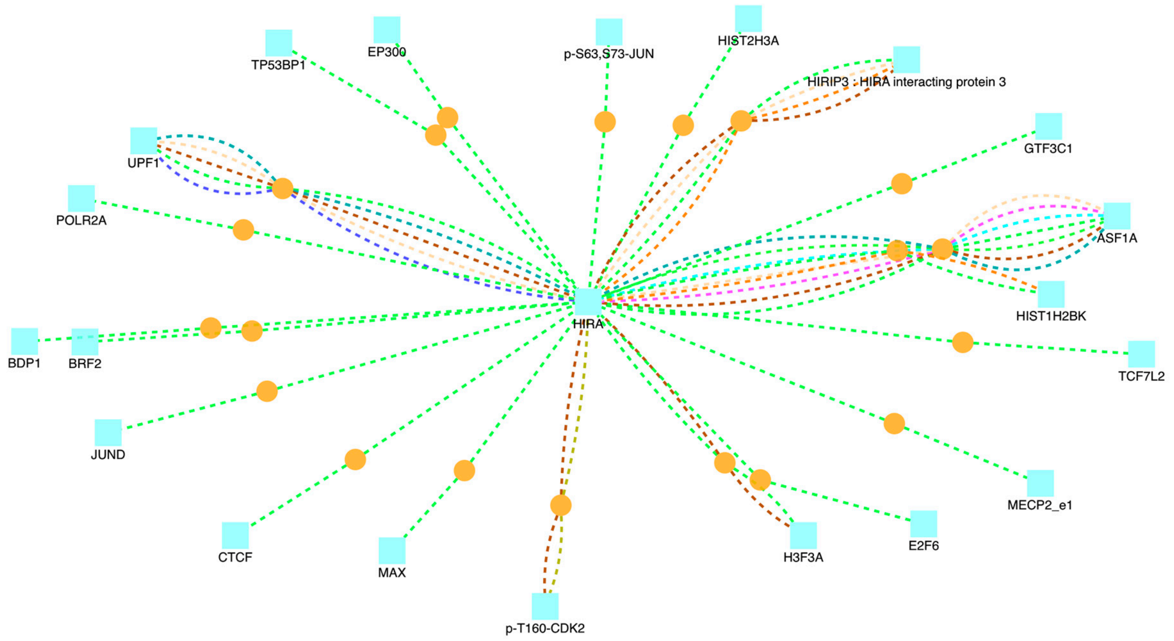

Figure S3. Visualization of HIRA protein interactions generated by ConsensusPath DB software.

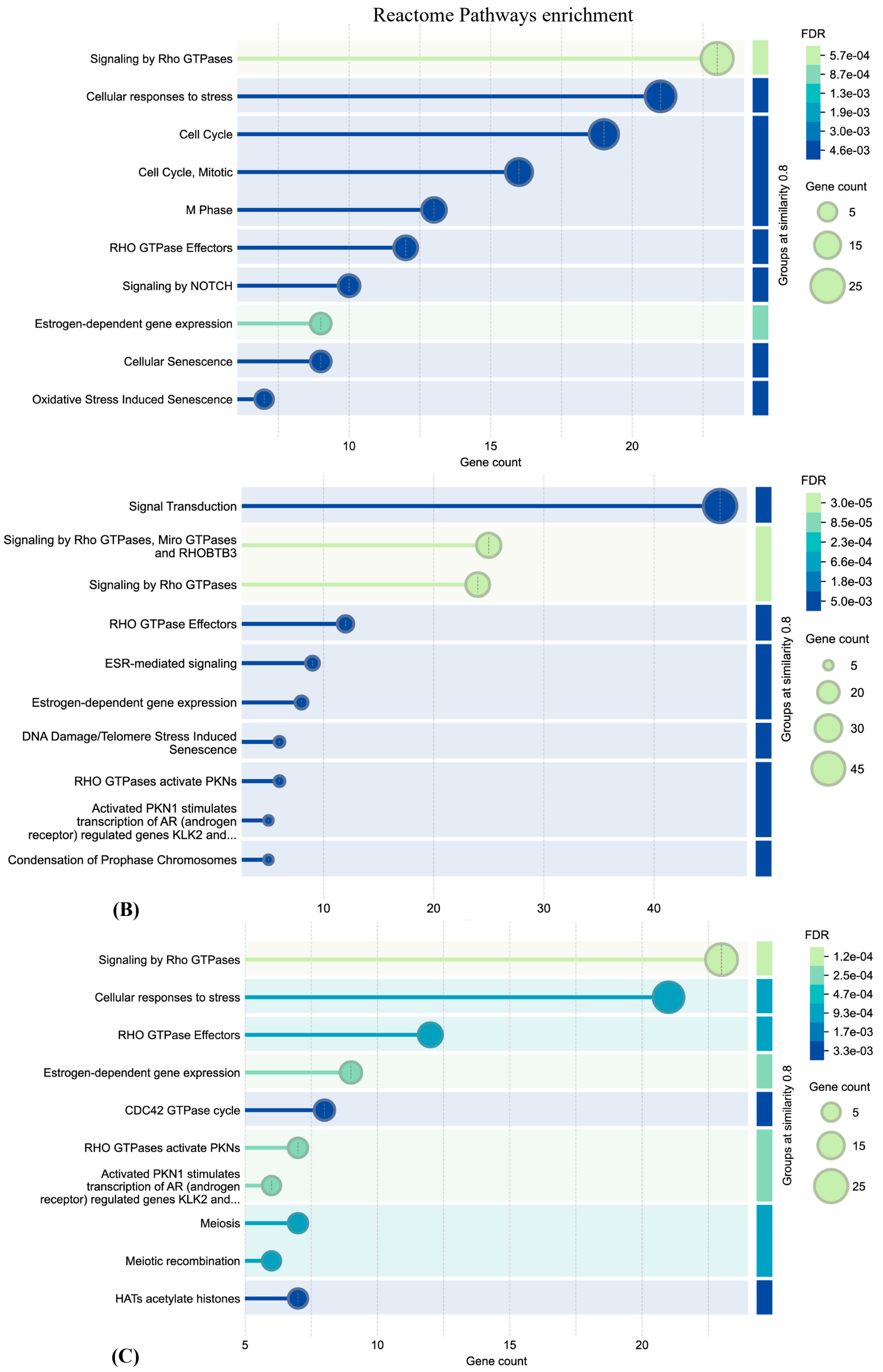

Figure S4. Functional Reactome pathway enrichment analysis of protein sets across different experimental conditions: (A) AFB1, (B) OTA, (C) AFB1+OTA. The color intensity reflects the False Discovery Rate (FDR), with darker shades indicating greater statistical significance. Circle size denotes the number of enriched proteins in each category.

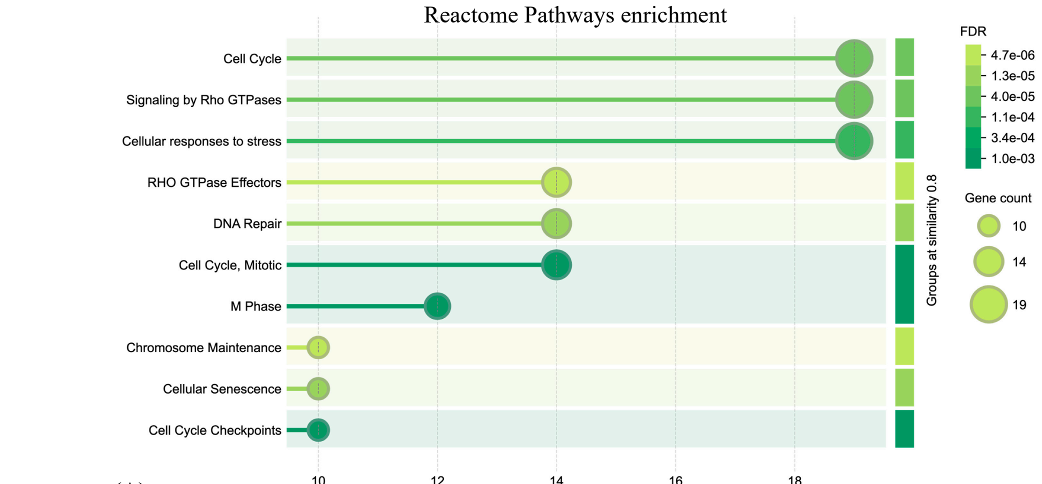

(A)

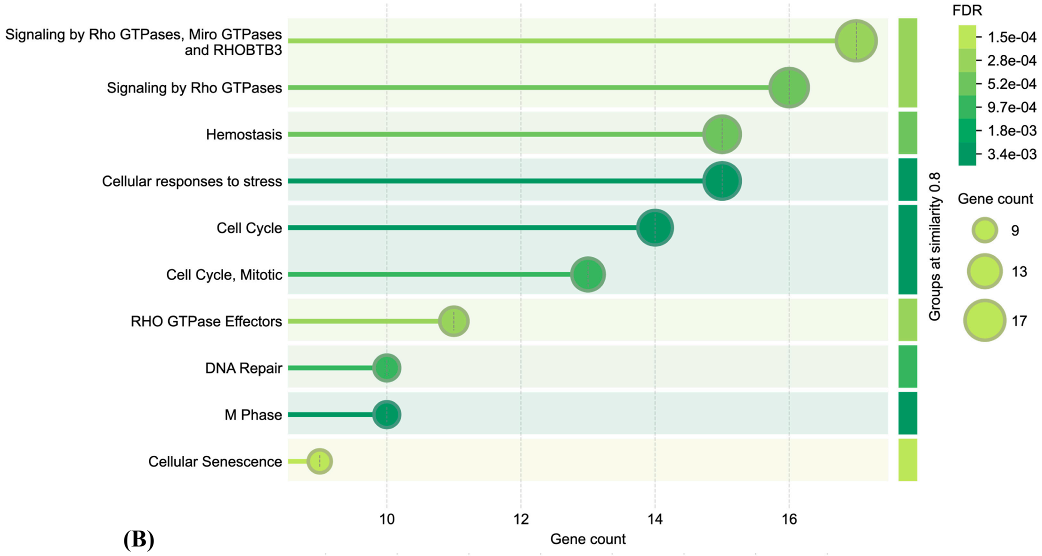

(B)

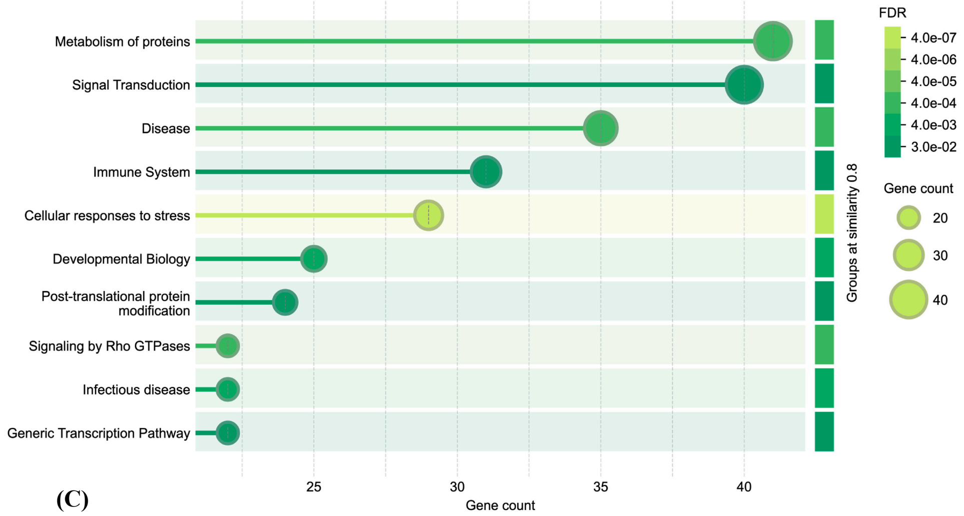

(C)

Figure S5. Functional Reactome pathway enrichment analysis of protein sets across different experimental conditions: (A) PID+AFB1, (B) PID+OTA, (C) PID+AFB1+OTA. The color intensity reflects the False Discovery Rate (FDR), with darker shades indicating greater statistical significance. Circle size denotes the number of enriched proteins in each category.

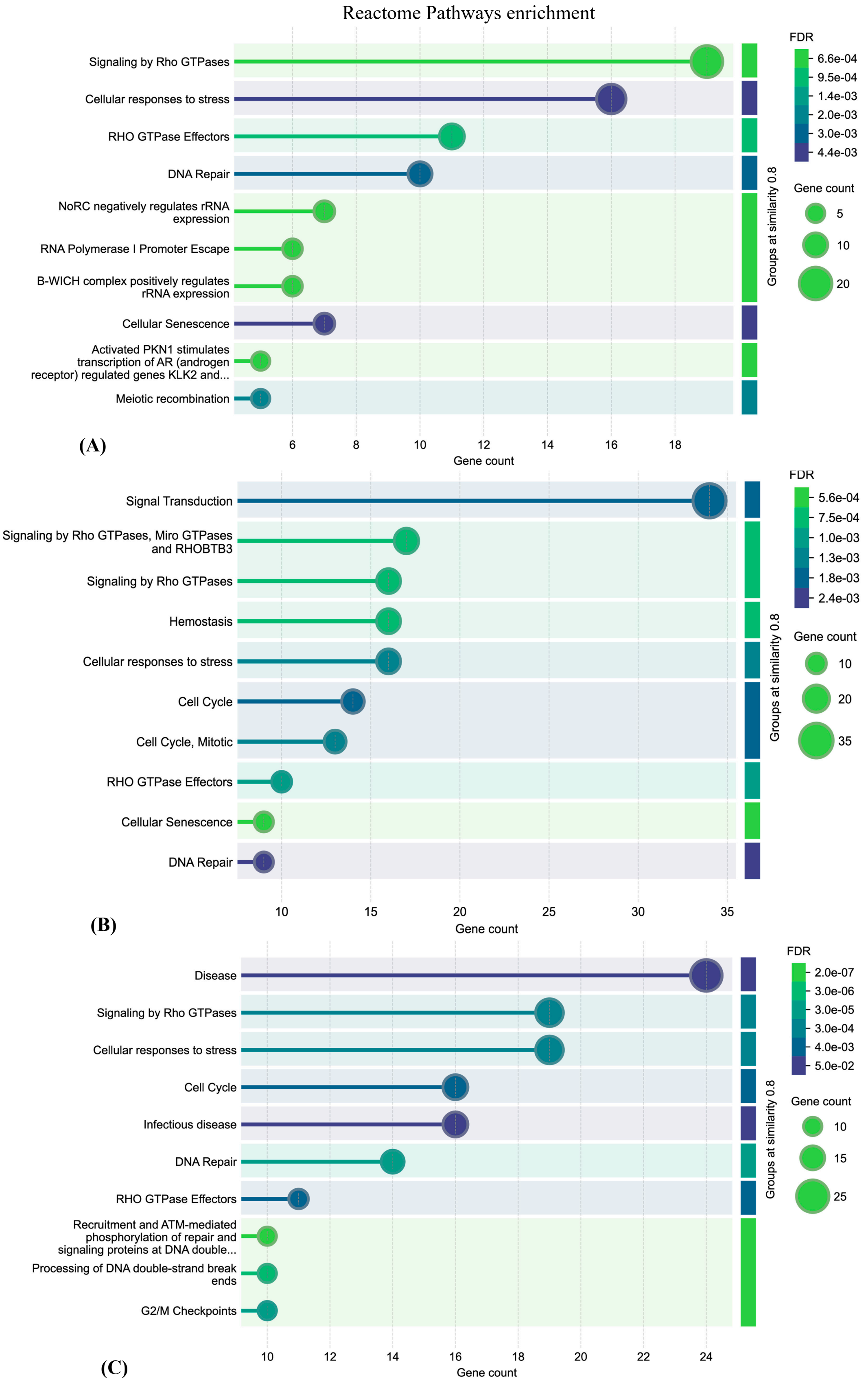

Figure S6. Functional Reactome pathway enrichment analysis of protein sets across different experimental conditions: (A) PID+FW+AFB1, (B) PID+FW+OTA, (C) PID+FW+AFB1+OTA. The color intensity reflects the False Discovery Rate (FDR), with darker shades indicating greater statistical significance. Circle size denotes the number of enriched proteins in each category.

Table S1. Phenolic compounds identified in intestinal digest of bread enriched with PID+FW by LC-MS/MS-QTOF.

Analyte	RT (min)	Elemental Composition	Adduct	Theoretical Mass (*m/z*)	Measured Mass (*m/z*)
p-Coumaric acid	2.43	$C_9H_8O_3$	[M-H]-	164.0473	163.0400
Sinapic acid	6.31	$C_{11}H_{12}O_5$	[M-H]-	224.0685	223.0611
Benzoic acid	2.47	$C_7H_6O_2$	[M-H]-	122.0368	121.0296
Lactic acid	0.46	$C_3H_6O_3$	[M-H]-	90.0316	89.0244
DL-3-Phenyllactic acid	4.94	$C_9H_{10}O_3$	[M-H]-	166.0630	165.0557
3-4-Dihydroxyhydrocinnamic	6.91	$C_9H_{10}O_4$	[M-H]-	182.0582	181.0508
Vanillic acid	4.71	$C_8H_8O_4$	[M-H]-	168.0423	167.0350
1-2-Dihydroxybenzene	3.42	$C_6H_6O_2$	[M-H]-	110.0368	109.0295
Vanillin	6.5	$C_8H_8O_3$	[M-H]-	151.0401	152.0477
Caffeic acid	6.31	$C_9H_8O_4$	[M-H]-	179.0352	180.0427
Ferulic acid	5.81	$C_{10}H_{10}O_4$	[M-H]-	194.0579	193.0506
Hydroxycinnamic acid	5.32	$C_9H_{10}O_2$	[M-H]-	195.0660	195.0662

Table S2. Concentration (µg/mL) and profile of carotenoids identified from intestinal digests of pumpkin (PID) and PID with fermented whey (FW).

Carotenoids concentration (µg/mL)						
**Bread**	**β-carotene**	**Lutein**	**Antheraxanthin**	**Violaxanthin**	**Zeaxanthin**	**β-cryptoxanthin**
PID	0.7364	0.0181	nd[1]	1.3309	0.0264	1.0241
PID+ AFB1	0.7091	0.0050	0.0001	0.9779	0.0067	0.6988
PID+OTA	0.5394	0.0036	nd	0.4978	0.0053	0.3627
PID+AFB1+OTA	0.2742	0.0036	0.0002	0.6654	0.0019	0.5470
PID+FW	1.8636	0.0180	nd	3.3676	nd	2.9880
PID+FW+AFB1	2.5152	0.0122	0.0054	3.0368	0.0054	2.3976
PID+FW+OTA	4.0000	0.0120	nd	3.3971	nd	2.3614
PID+FW+AFB1+OTA	2.2879	0.0086	0.0580	2.7426	0.0580	1.8554

[1]nd, not detected
