# Supplementary figures and images for: Deepening the Modulatory Activity of Bioactive Compounds Against AFB1- and OTA-Induced Neuronal Toxicity Through a Proteomic Approach
Source: Antioxidants (Basel). 2025 May 9;14(5):571. doi: 10.3390/antiox14050571 (PMC12108279; doi:10.3390/antiox14050571)

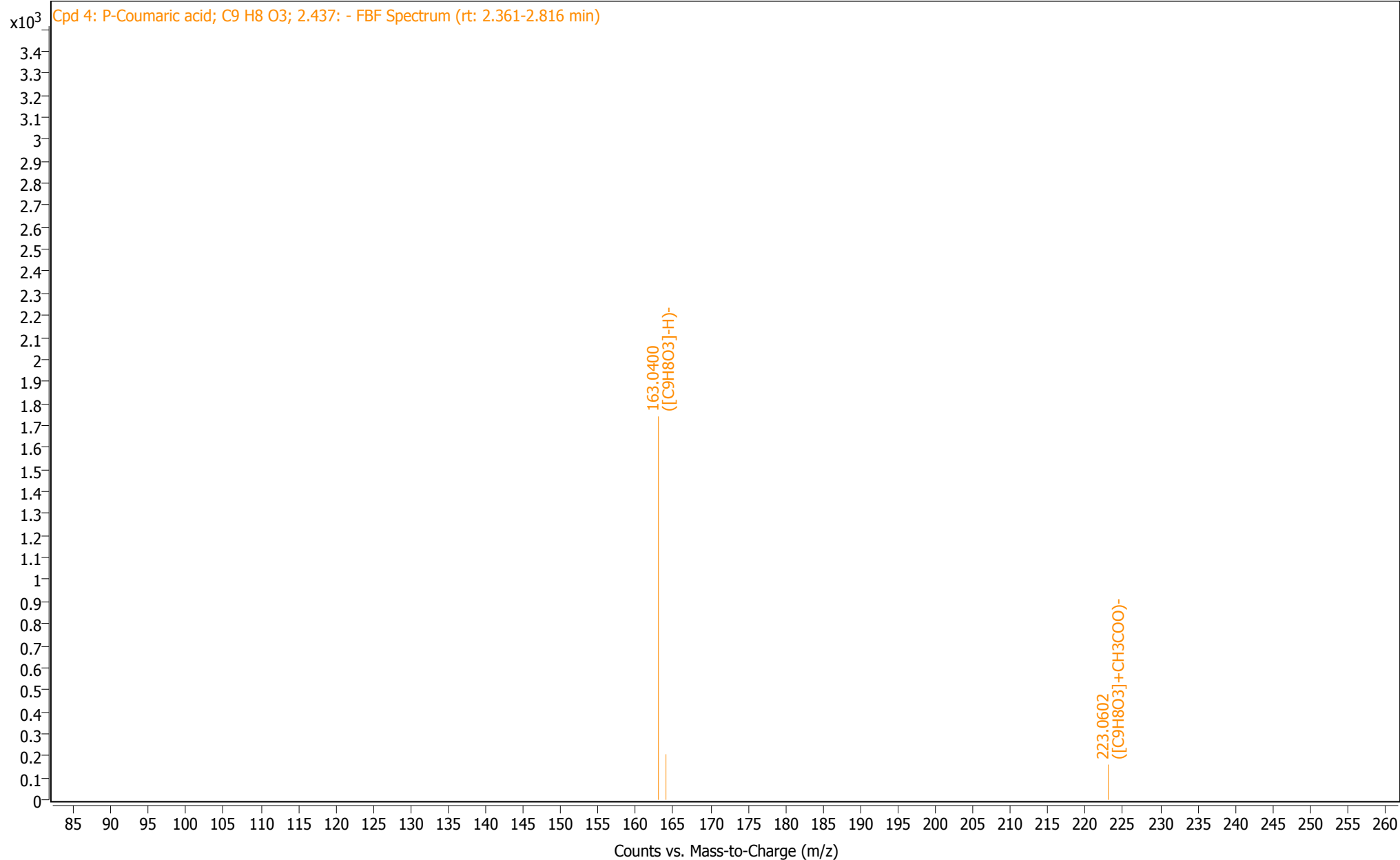

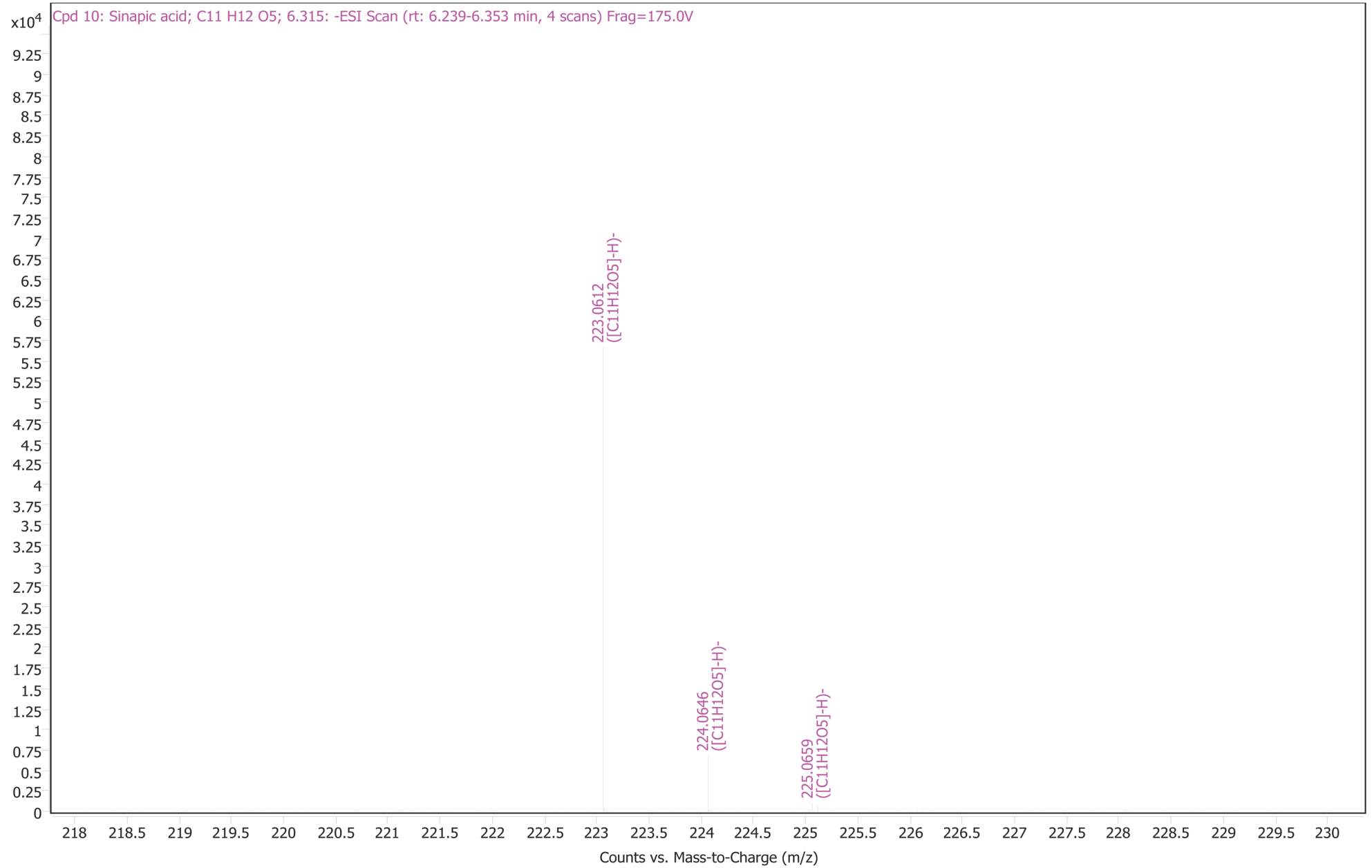

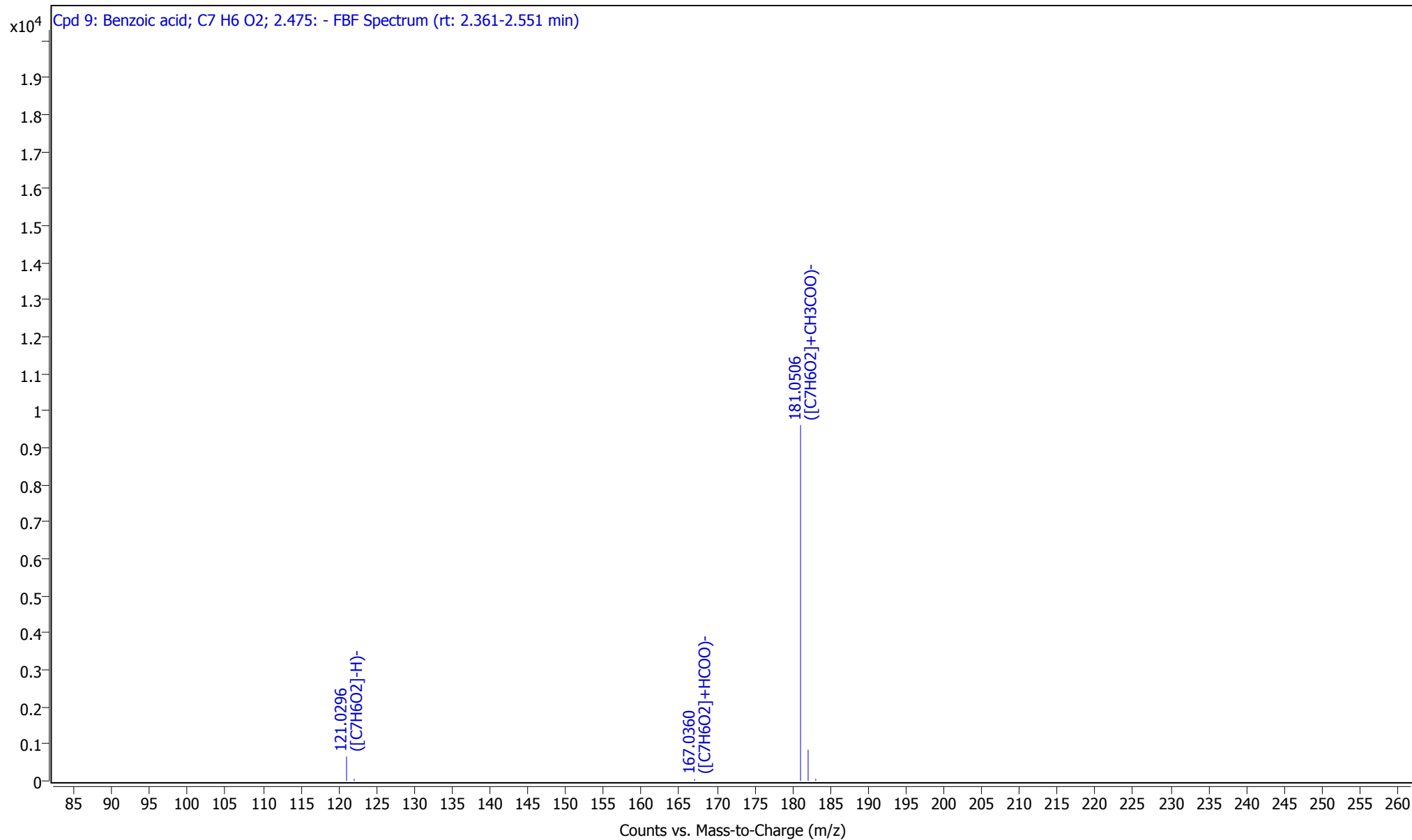

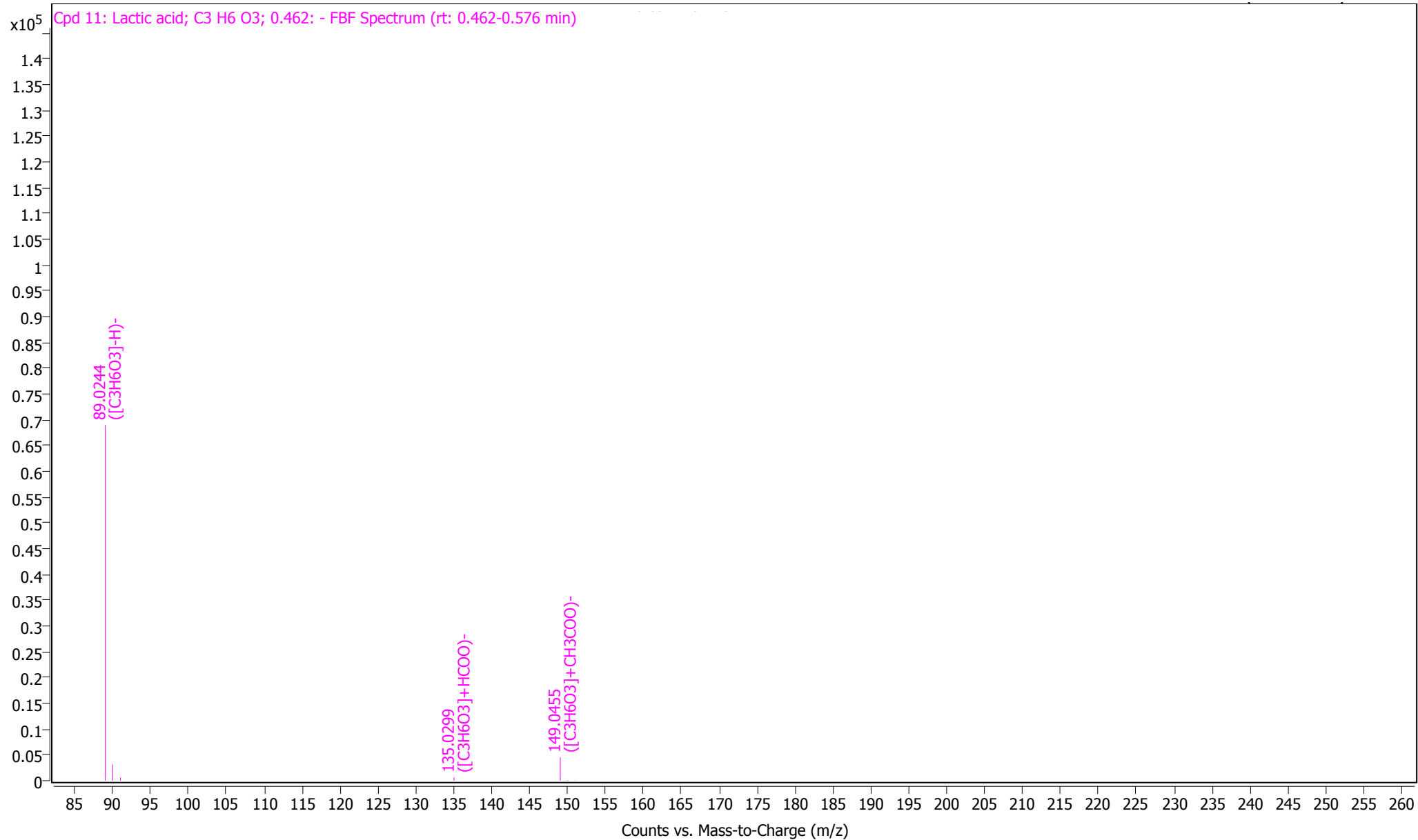

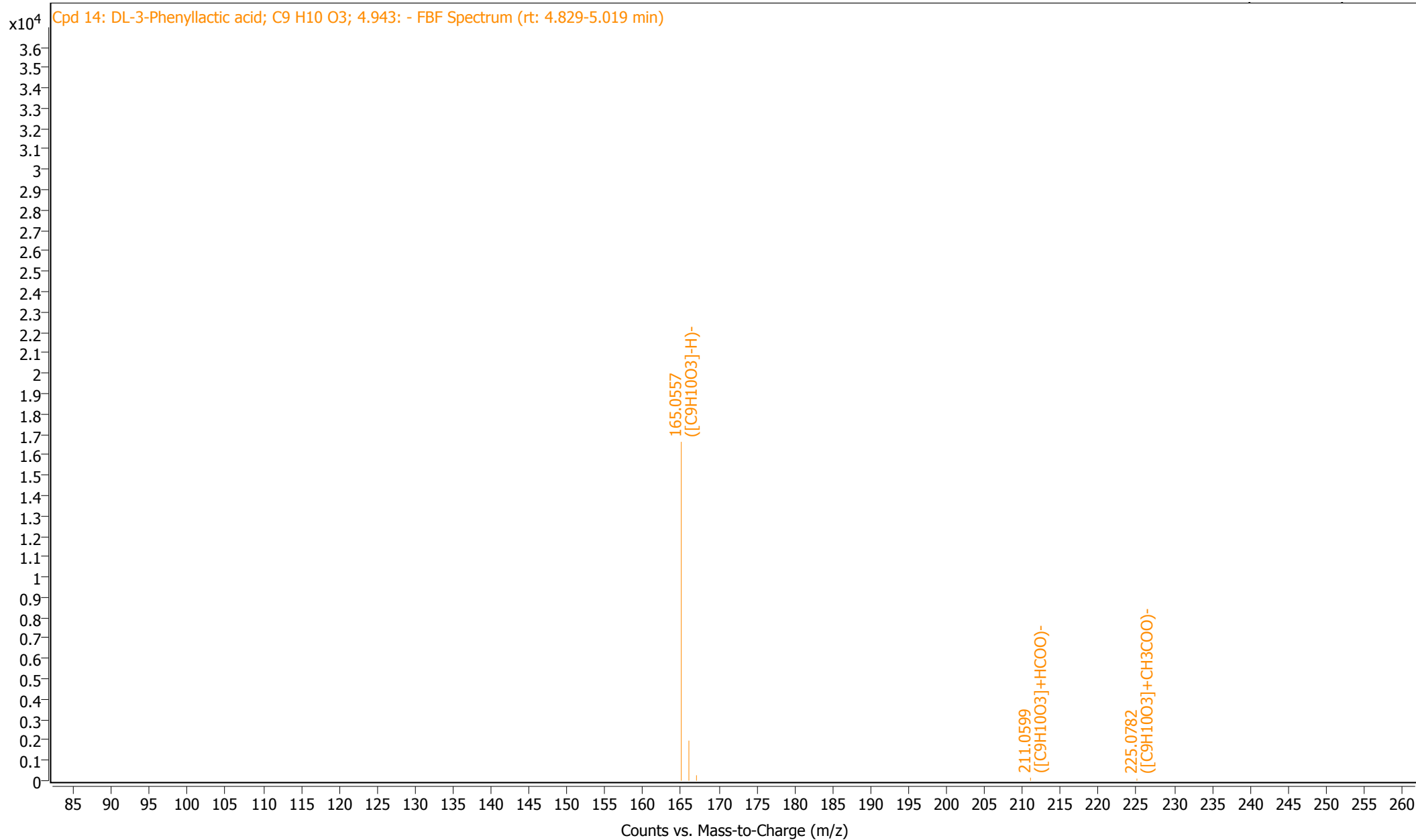

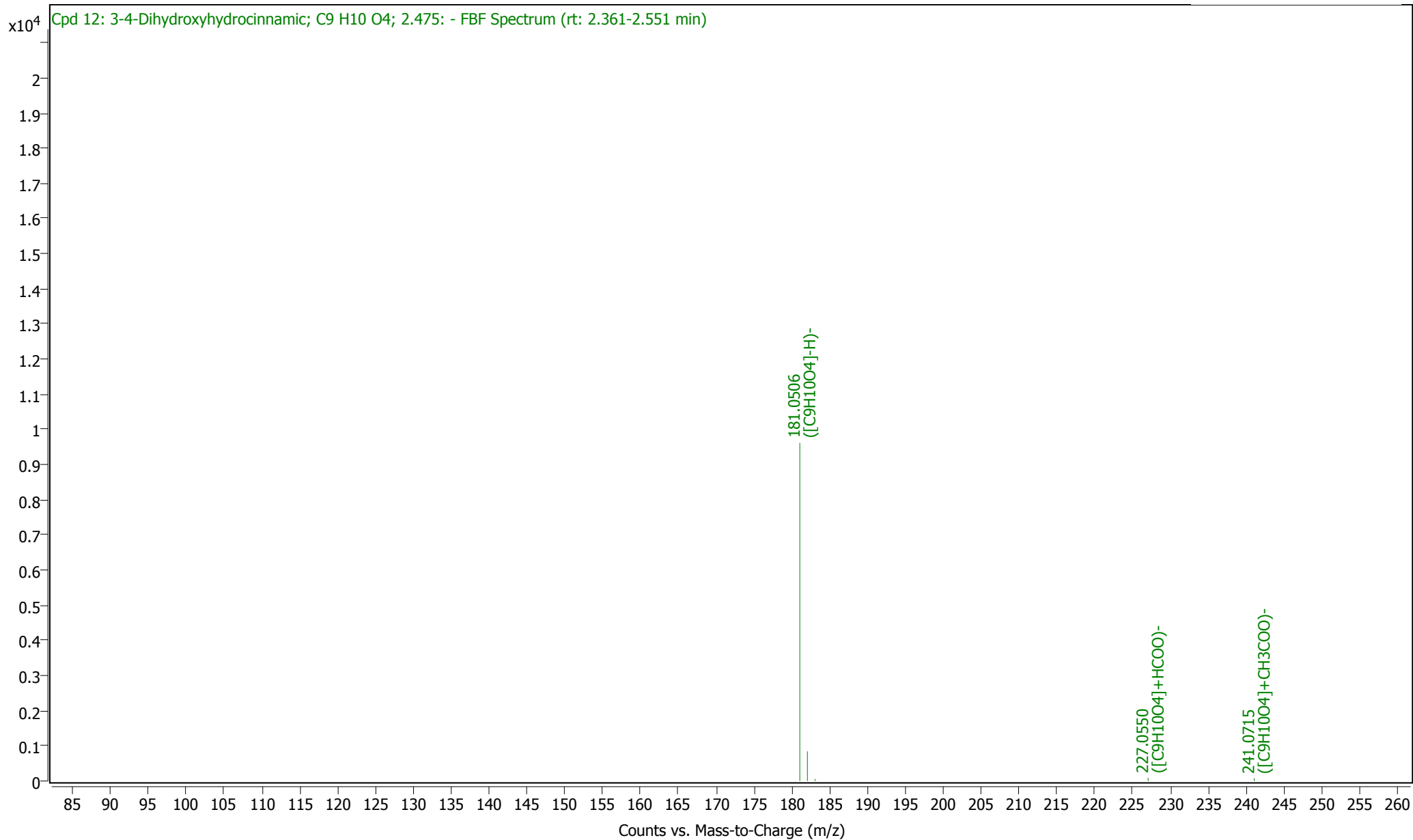

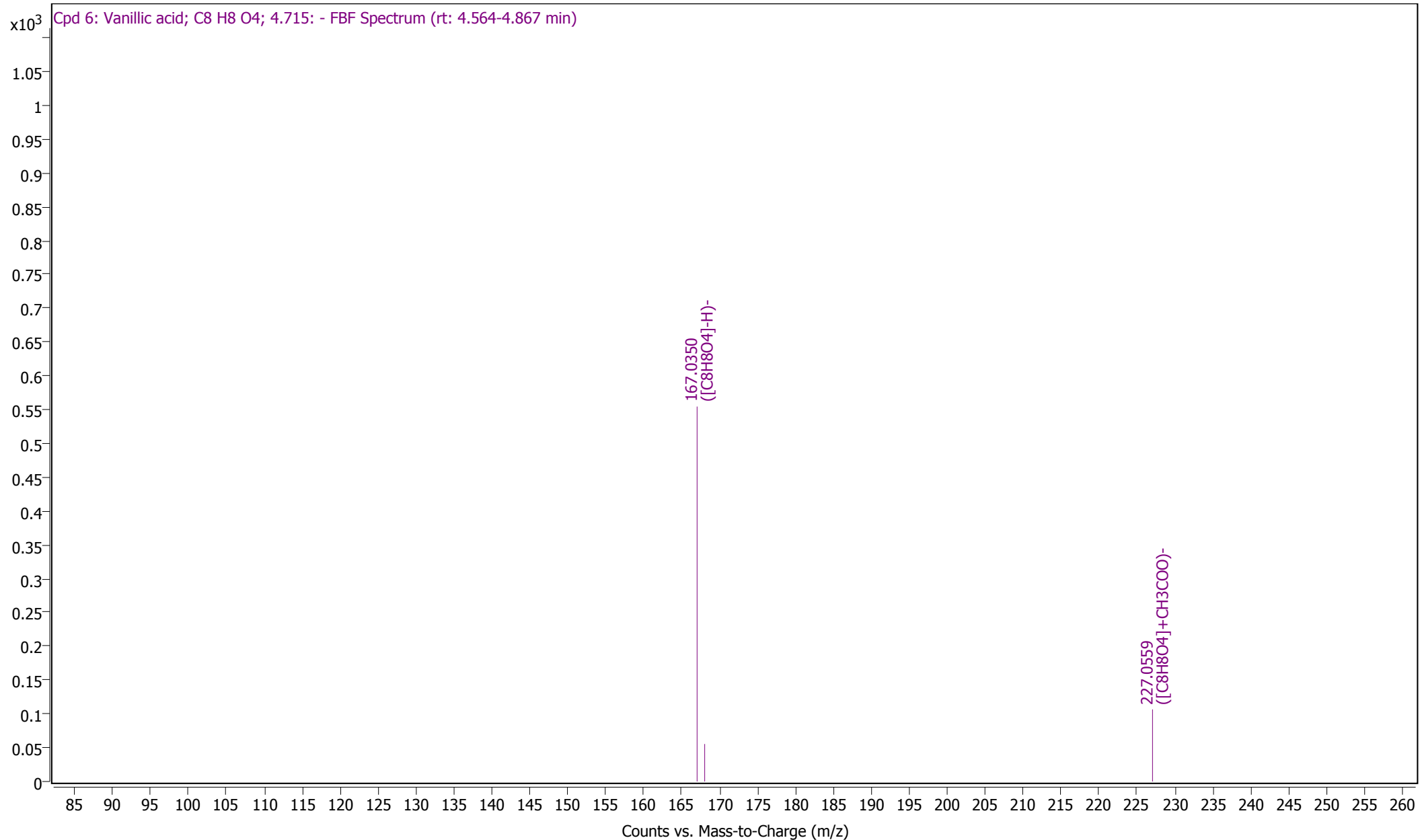

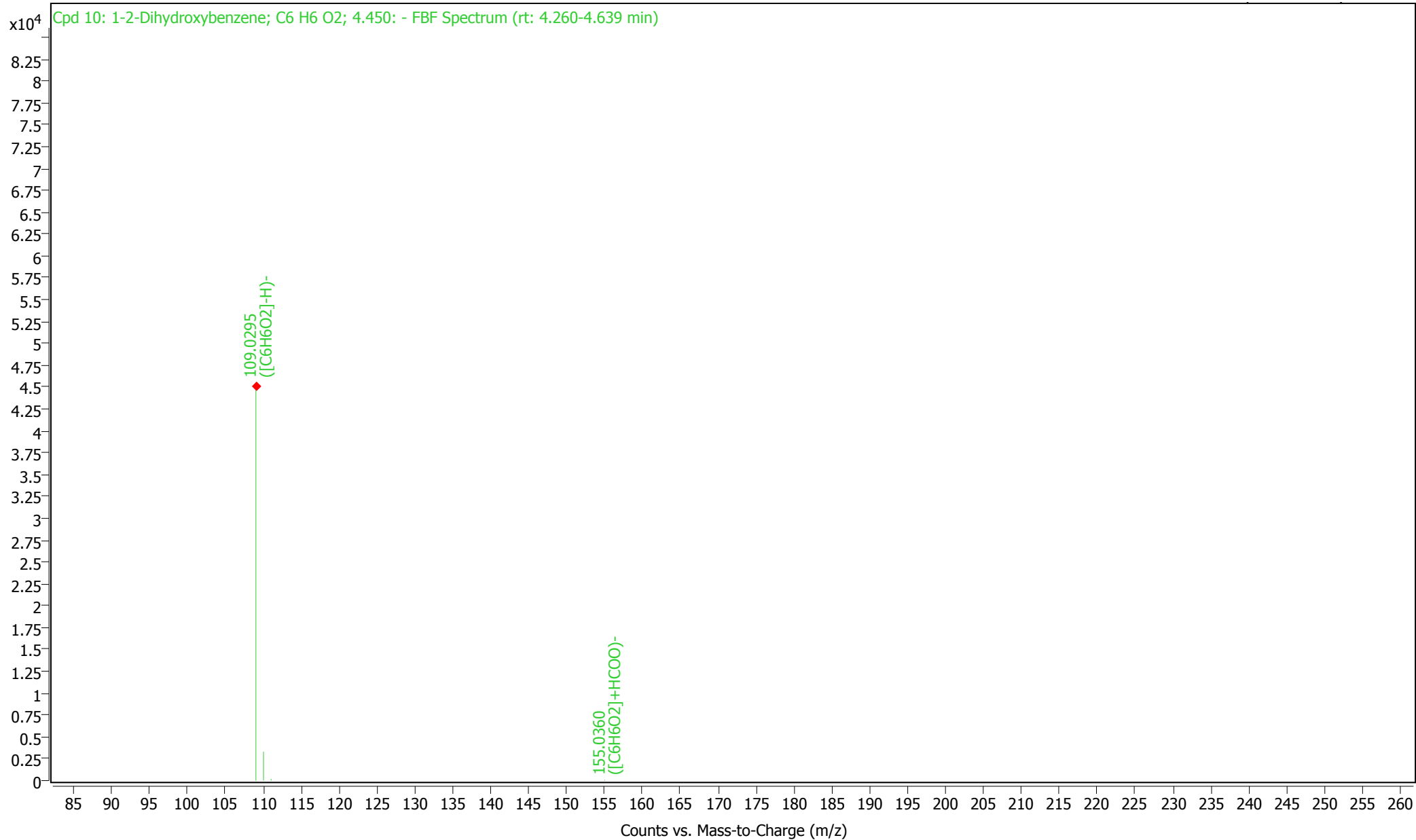

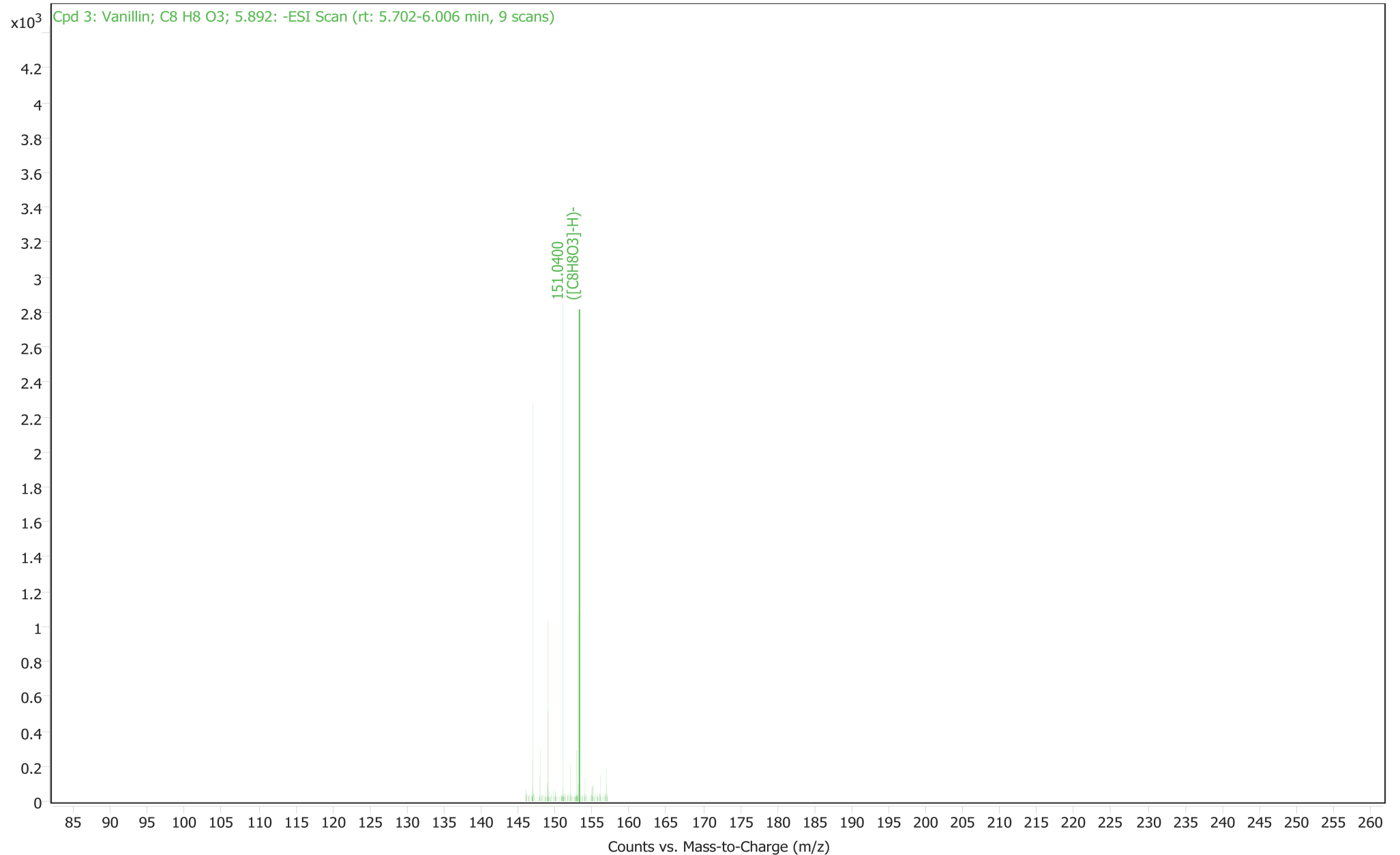

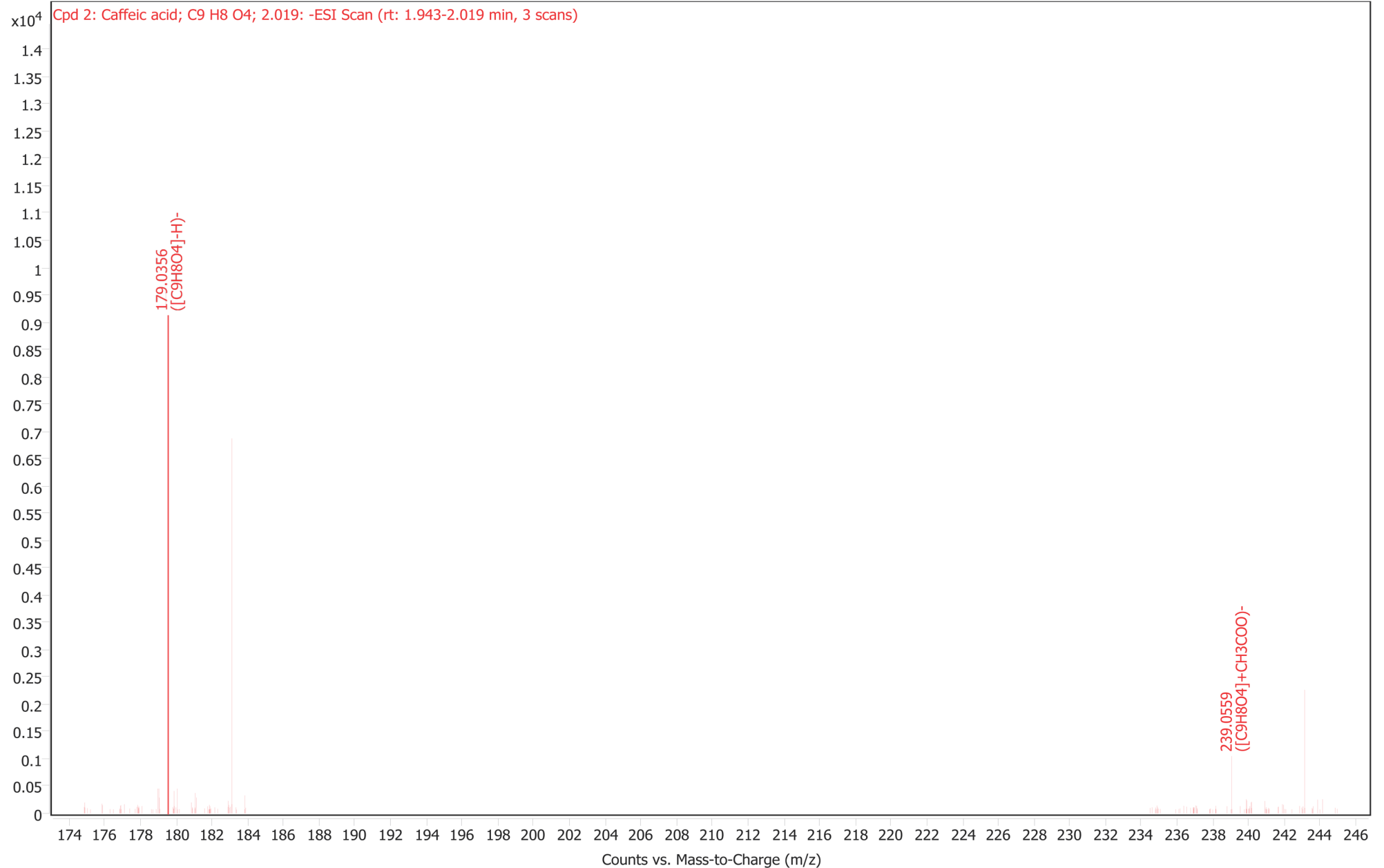

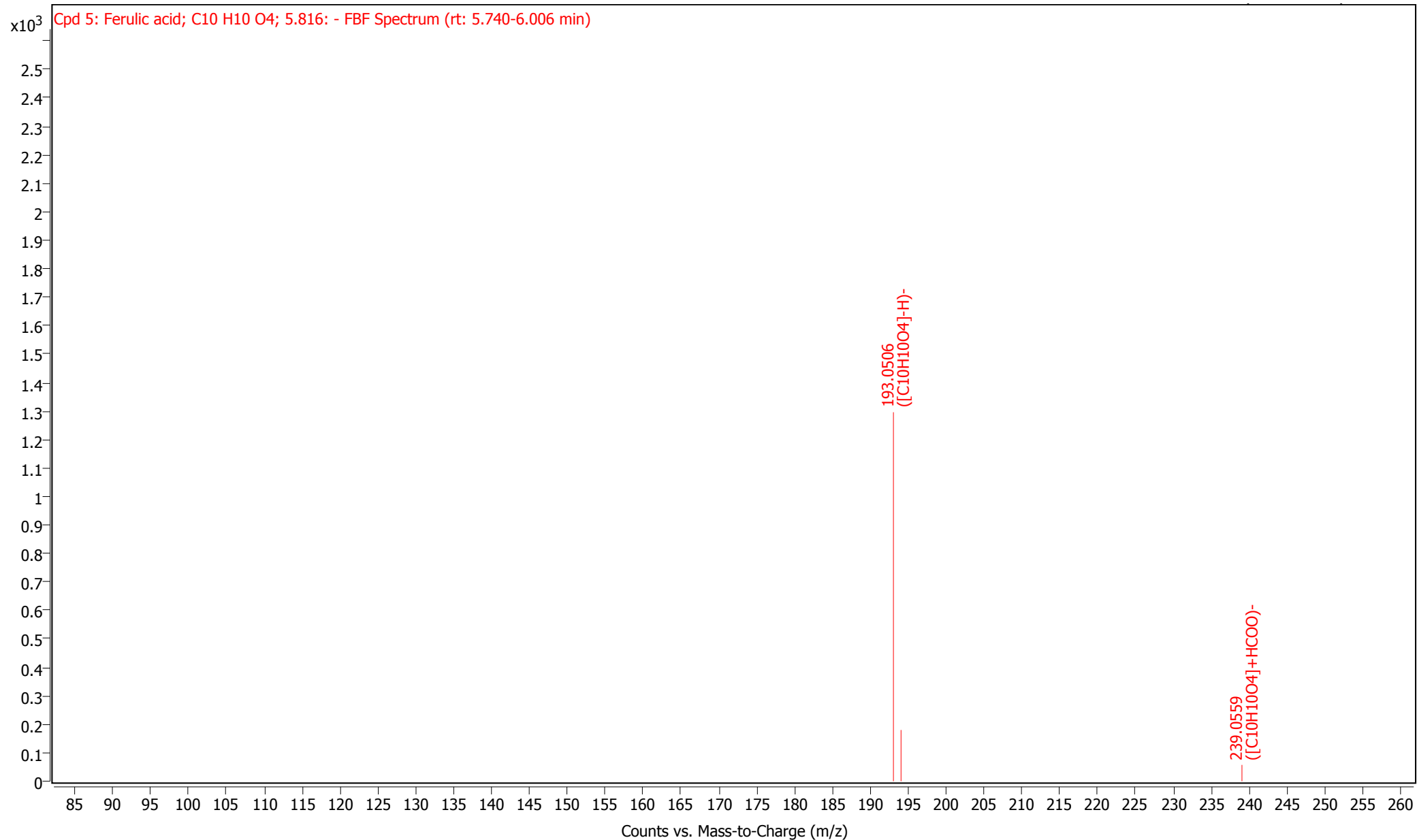

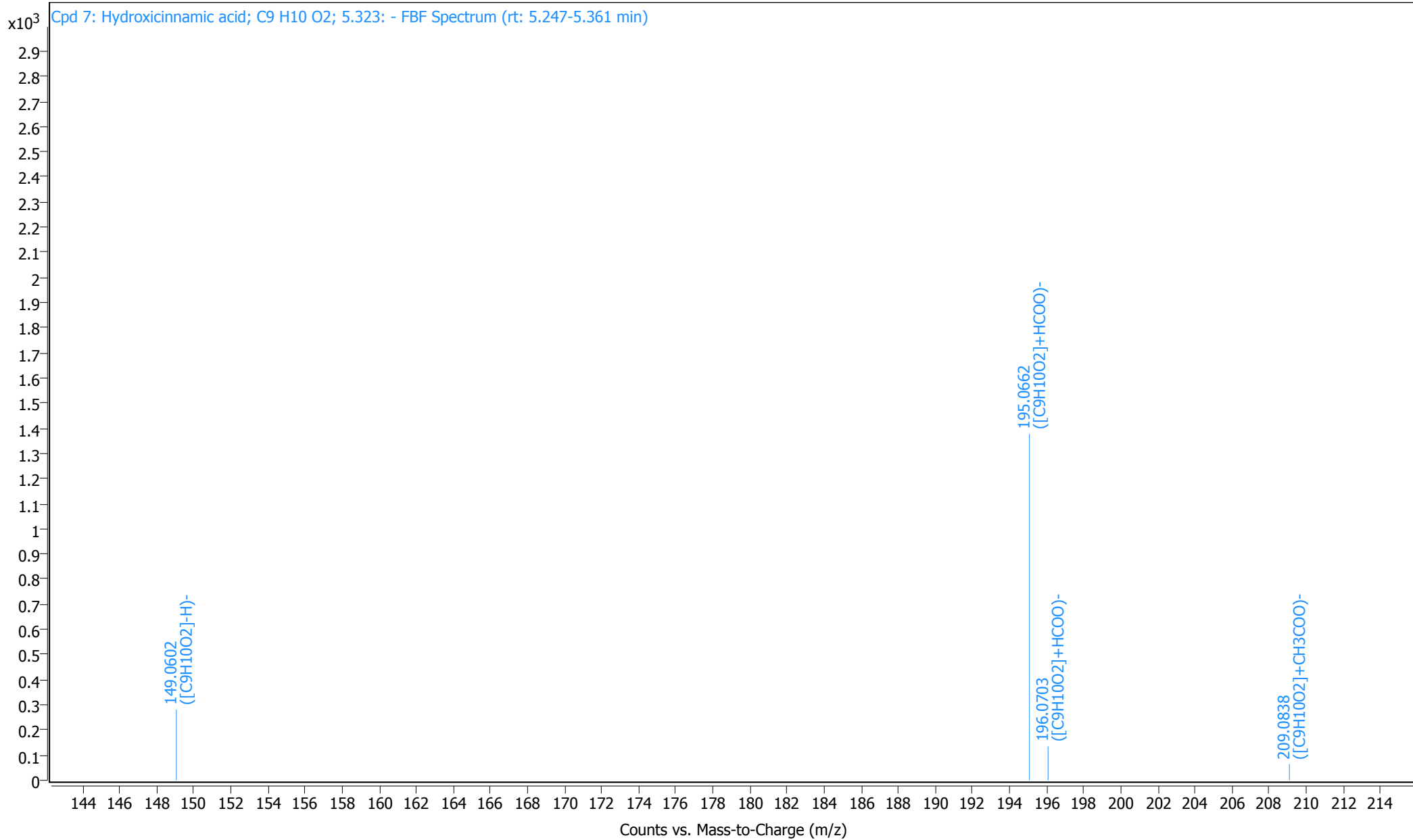

Supplement: Supplementary file 1 [file antioxidants-14-00571-s001.zip › Supplementary material S2.pdf]
